# Diseases Caused by Mutations in Mitochondrial Carrier Genes *SLC25*: A Review

**DOI:** 10.3390/biom10040655

**Published:** 2020-04-23

**Authors:** Ferdinando Palmieri, Pasquale Scarcia, Magnus Monné

**Affiliations:** 1 Department of Biosciences, Biotechnologies and Biopharmaceutics, Laboratory of Biochemistry and Molecular Biology, University of Bari Aldo Moro, via E. Orabona 4, 70125 Bari, Italy; pasquale.scarcia@uniba.it; 2 Department of Sciences, University of Basilicata, via Ateneo Lucano 10, 85100 Potenza, Italy

**Keywords:** disease, error of metabolism, mitochondrial carrier, mitochondrial carrier disease, mitochondrial disease, mitochondrial transporter, membrane transport, mutation, SLC25.

## Abstract

In the 1980s, after the mitochondrial DNA (mtDNA) had been sequenced, several diseases resulting from mtDNA mutations emerged. Later, numerous disorders caused by mutations in the nuclear genes encoding mitochondrial proteins were found. A group of these diseases are due to defects of mitochondrial carriers, a family of proteins named solute carrier family 25 (SLC25), that transport a variety of solutes such as the reagents of ATP synthase (ATP, ADP, and phosphate), tricarboxylic acid cycle intermediates, cofactors, amino acids, and carnitine esters of fatty acids. The disease-causing mutations disclosed in mitochondrial carriers range from point mutations, which are often localized in the substrate translocation pore of the carrier, to large deletions and insertions. The biochemical consequences of deficient transport are the compartmentalized accumulation of the substrates and dysfunctional mitochondrial and cellular metabolism, which frequently develop into various forms of myopathy, encephalopathy, or neuropathy. Examples of diseases, due to mitochondrial carrier mutations are: combined D-2- and L-2-hydroxyglutaric aciduria, carnitine-acylcarnitine carrier deficiency, hyperornithinemia-hyperammonemia-homocitrillinuria (HHH) syndrome, early infantile epileptic encephalopathy type 3, Amish microcephaly, aspartate/glutamate isoform 1 deficiency, congenital sideroblastic anemia, Fontaine progeroid syndrome, and citrullinemia type II. Here, we review all the mitochondrial carrier-related diseases known until now, focusing on the connections between the molecular basis, altered metabolism, and phenotypes of these inherited disorders.

## Contents

1. Introduction
2. Characteristic features of mitochondrial carriers (MCs)
3. Diseases caused by mutations in MCs
  3.1. General information
  3.2. SLC25A1 (citrate carrier, CIC) deficiency
  3.3. SLC25A3 (phosphate carrier, PiC) deficiency
  3.4. SLC25A4 (ADP/ATP carrier 1, AAC1) deficiency
  3.5. SLC25A10 (dicarboxylate carrier, DIC) deficiency
  3.6. SLC25A12 (aspartate-glutamate carrier 1, AGC1, aralar) deficiency
  3.7. SLC25A13 (aspartate-glutamate carrier 2, AGC2, citrin) deficiency
  3.8. SLC25A15 (ornithine carrier 1, ORC1) deficiency
  3.9. SLC25A16 deficiency
  3.10. SLC25A19 (thiamine pyrophosphate carrier, TPC) deficiency
  3.11. SLC25A20 (carnitine-acylcarnitine carrier, CAC) deficiency
  3.12. SLC25A21 (oxodicarboxylate carrier, ODC) deficiency
  3.13. SLC25A22 (glutamate carrier 1, GC1) deficiency
  3.14. SLC25A24 (ATP-Mg2^+^/phosphate carrier 1, APC1) deficiency
  3.15. SLC25A26 (S-adenosylmethionine carrier, SAMC) deficiency
  3.16. SLC25A32 deficiency
  3.17. SLC25A38 (glycine carrier, GlyC) deficiency
  3.18. SLC25A42 (CoA and PAP carrier) deficiency
  3.19. SLC25A46 deficiency
4. Multigenic diseases with mutations in MCs
5. Concluding remarks

## 1. Introduction

Mitochondrial diseases are caused by mutations in either the mitochondrial or nuclear genome. Out of about 1500 proteins comprising the mitochondrial proteome, only 13 proteins, plus 2 ribosomal RNAs and 22 transfer RNAs, are encoded by the mitochondrial DNA (mtDNA) and all the rest by the nuclear DNA. Until now, more than 350 mutations have been identified as causing mitochondrial disorders [1,2]. The mutations in the nuclear genes are inherited in a Mendelian fashion, whereas those of the multi-copy mtDNA follow a maternal pattern of inheritance.

Mitochondrial diseases are a very heterogeneous group of pathologies in many aspects. The clinical features of these disorders manifest differently in various tissues and cell types but typically involve the brain, muscle, heart, kidney, liver, and retina—organs with high energy demands [3]. The age of onset of these disorders ranges from very early in the neonatal period to late in adulthood, and the symptoms are sometimes triggered by environmental factors and may occur suddenly or develop progressively.

The disease-causing mutations in the mtDNA primarily involve core subunits of the respiratory chain and oxidative phosphorylation complexes as well as gene products involved in mitochondrial translation. These defects give rise to, for example, Leigh syndrome, LHON (Leber hereditary optic neuropathy), MELAS (mitochondrial myopathy, encephalopathy, lactic acidosis, and stroke-like episodes syndrome), MERRF (myoclonic epilepsy with ragged fibers), and NARP (neurogenic muscle weakness ataxia and retinitis pigmentosa) [3,4]. The pathogenic nuclear mutations affect mitochondrial proteins involved in many important processes, such as the respiratory chain, oxidative phosphorylation, metabolism, cofactor biosynthesis, transport, replication, transcription, translation, iron homeostasis, cell signalling, organelle dynamics, protein biogenesis, and complex assembly [5]. Among the mitochondrial diseases caused by mutations in nuclear genes are: Friedreich’s ataxia, dominant optic atrophy, Barth syndrome, Rett syndrome, spastic paraplegia, coenzyme Q10 deficiency, and progressive external ophthalmoplegia [3,4]. Furthermore, mutations in both nuclear and mitochondrial DNA play a role in diabetes, obesity, autism, Alzheimer’s disease, Parkinson’s disease, Huntington’s disease, cancer, and aging [6].

This review focusses on the subgroup of mitochondrial diseases that are caused by mutations in the genes encoding members of the mitochondrial carrier family SLC25. Particular emphasis is given to the location and relevance of the mutations within the structure of the transporters and to the pathogenic mechanisms underlying the symptoms characteristic of the diseases.

## 2. Characteristic Features of Mitochondrial Carriers

Mitochondrial carriers (MCs) constitute a protein family also called the solute carrier family 25 (SLC25). It is the largest of the transporter families, its 53 human members are named from SLC25A1 to SLC25A53, and it is widely distributed in all eukaryotes [7,8,9]. The sequences of MCs contain three repeated domains, each of which consists of about 100 residues and includes two transmembrane segments that are linked by a signature motif sequence (SMS): PX[DE]XX[KR]X[KR]X20–30[DE]GXXXX[WYF][KR]G (PROSITE PS50920, PFAM PF00153 and IPR00193) [10,11]. These highly conserved sequence features are specific for MCs and have been used for the identification of family members in genomic sequences. The substrates transported by most MCs in *Homo sapiens*, *Saccharomyces cerevisiae* and *Arabidopsis thaliana*, have been identified by the EPRA method: recombinant expression of MC genes, purification of the proteins, and their reconstitution into liposomes that are subjected to direct transport assays [12,13,14]. By this means, it has been shown that MCs transport a great variety of substrates: nucleotides (e.g., ATP, ADP and dNTPs), cofactors (e.g., thiamine pyrophosphate, S-adenosylmethionine (SAM), coenzyme A (CoA) and NAD^+^), metabolites with carboxylic groups (e.g., malate, 2-oxoglutarate and citrate), amino acids (e.g., aspartate, glutamate, ornithine and arginine), and inorganic ions (e.g., phosphate and sulfate). The transport of the substrates across the inner mitochondrial membrane, i.e., between the intermembrane space/cytoplasm and the mitochondrial matrix and vice versa, is mediated by specific MCs. Consequently, MCs play numerous important roles in energy, nucleotide, fatty acid, and amino acid metabolism as well as in providing cofactors for enzymes and precursors for replication, transcription, and translation in mitochondria. The great majority of MCs catalyze an exchange (antiport) of substrates, but some also catalyze the unidirectional transport (uniport) of substrates, like the phosphate carrier, carnitine carrier, and UCP1 [9,15]. MC superfamily members that have the same or very similar substrate specificity form subfamilies, which are also characterized by distinctive structural and transport features [16,17]. At variance with all the other MCs, the aspartate-glutamate and ATP-Mg/phosphate carrier subfamilies have N-terminal extensions containing EF-hand Ca^2+^-binding motifs and are regulated by Ca^2+^ [18,19,20]. It is noteworthy that not all the characterized MCs are localized in the mitochondrial inner membrane; some are located in other organelles such as chloroplasts and peroxisomes [16,17]. MCs are synthesized on cytosolic ribosomes and imported usually into the inner mitochondrial membrane. Unlike most nuclear-encoded mitochondrial proteins, the targeting information is contained in their mature sequence. Only a few of them possess short cleavable presequences, which, however, are not essential for import, although those of phosphate and citrate carriers improve correct targeting [21,22,23,24].

In the atomic 3D-structures of the only MC family member determined until now, the ADP/ATP carrier (AAC), the six transmembrane segments form a bundle of α-helices (H1-H6) around a central cavity, which constitutes the substrate translocation pore, whereas the 20–30 less conserved residues in the middle of the SMSs form three α-helices almost parallel to the membrane on the matrix side [25,26,27]. Two conformations of AAC are trapped by the inhibitors carboxyatractyloside and bongkrekic acid that lock the protein with the substrate translocation pore alternatively closed towards the matrix or intermembrane space by a matrix (m) gate or a cytosolic (c) gate, respectively [28,29]. These two conformations (named c-state and m-state) are thought to correspond approximately to the carrier (in the absence of the inhibitors) that is open to receive the substrate from the intermembrane space or from the mitochondrial matrix, respectively. In the c-state, the matrix gate is formed by the three initial stretches of the SMS (PX[DE]XX[KR]), where the prolines kink H1, H3, and H5 and the two charged residues are involved in salt-bridges interconnecting these three helices. In the m-state, this salt-bridge relay is broken and a similar (but less conserved) salt-bridge network is formed by the c-gate residues [DE]XX[KR] located close to the intermembrane space on H2, H4, and H6. The structures [25,26,27] strengthen the hypothesized “single binding center-gating pore” transport mechanism for MCs, which was initially deduced from studies of the inhibitors carboxyatractyloside and bongkrekic acid and their competition with the transported substrates of AAC [28,29]. As indicated by the name of this mechanism there is a single substrate binding site centrally located in the pore of the protein, and its access to the substrate is regulated by the alternating opening and closing of the c- and m-gates. It is believed that the binding of the substrate to the active site when entering from the open side of the pore (either from the outside or matrix side) triggers conformational changes leading to the closure of the open gate, the opening of the opposite closed gate and the release of the substrate on the opposite side of the membrane with respect to where it entered the carrier; then another (or the same) substrate is translocated in the opposite direction to complete the exchange reaction. The central binding site has been suggested to be determined by residues of H2, H4, and H6 located in positions called contact points I, II, and III, respectively, approximately at the same level in the middle of the membrane [30,31,32]. Indeed, the properties of the residues in contact point II co-variate with the kind of substrate; the motifs G[IVLM], R[QHNT] and R[DE] are conserved at this location in carriers for nucleotides, carboxylates, and amino acids, respectively. The residues in the other contact points and in their proximity contribute to the particular substrate specificity of each MC [17,30,33,34].

## 3. Diseases Associated with Mutations in MCs

### 3.1. General Information

Since 1997, mutations in 18 different MCs have been reported to cause diseases (Table 1) [35,36,37]. They are all rare diseases (errors of metabolism) and, with a few exceptions, follow a recessive pattern of inheritance. At the biochemical level, these MC mutations give rise to the reduction or elimination of their respective substrate transport, which leads to accumulating metabolites and dysfunctional cellular metabolism. In common with other mitochondrial diseases, that are caused by mutations in mitochondrial proteins, many of the MC-associated pathologies often manifest with different forms of myopathy, encephalopathy, and neuropathy, and a few of them present loss of mitochondrial DNA as a secondary effect [38]. When MCs directly linked to oxidative phosphorylation (i.e., the ADP/ATP carrier and the Pi carrier) are altered, the organ-specific symptoms are due to the activities of heart, muscle, and other tissues requiring particularly high energy demands and therefore being highly dependent on mitochondrial function. Otherwise, the symptomatology of the MC-related diseases depends on several factors such as the specific metabolism affected, its relevance in specific tissues, the tissue distribution of the affected carrier, and the presence in the same tissues of isoforms or other MCs that can compensate, at least partly, the function(s).

**Figure 1:**
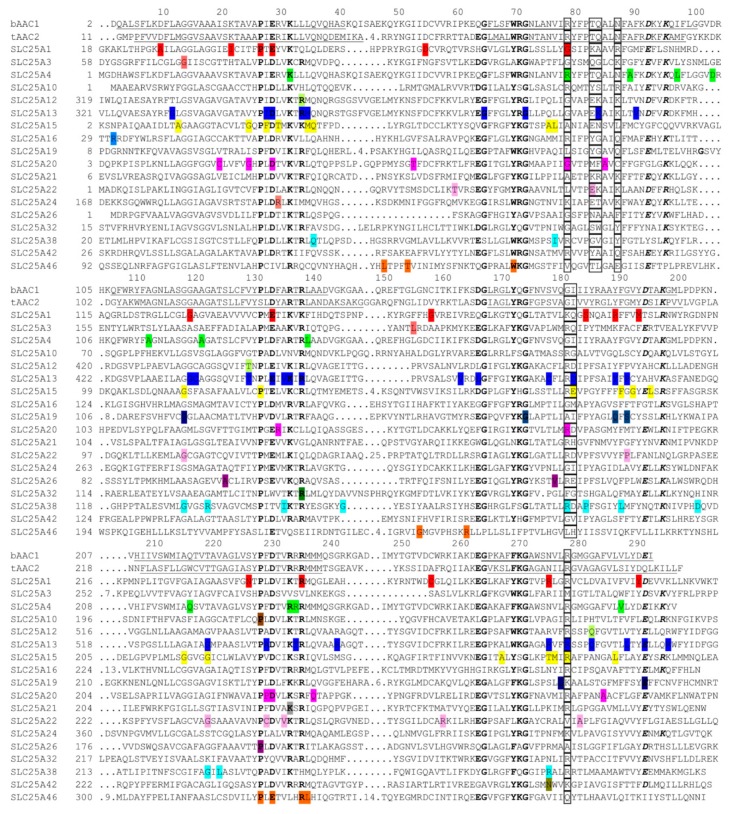
Multiple sequence alignment of mitochondrial carriers showing the position of disease-causing point mutations. The protein sequences of the 18 MCs found to have disease-causing point mutations are aligned against the sequences of bovine AAC1 (bAAC1 with numbering) and *Thermothelomyces thermophila* AAC2 (tAAC2) whose 3D-structures have been determined (Figure 2). Sequences of the transmembrane helices are underlined, the conserved residues of the SMSs are in bold, the charged residues of the cytoplasmic gate are in italics, and the contact points I, II, and III residues on the first, second, and third row of the alignment, respectively, are boxed. The numbers inside the alignment indicate missing residues. One color for each MC indicates the positions of the single point mutations associated with disease, and the top color in each position in the alignment corresponds to the colored positions in Figure 2.

**Figure 2:**
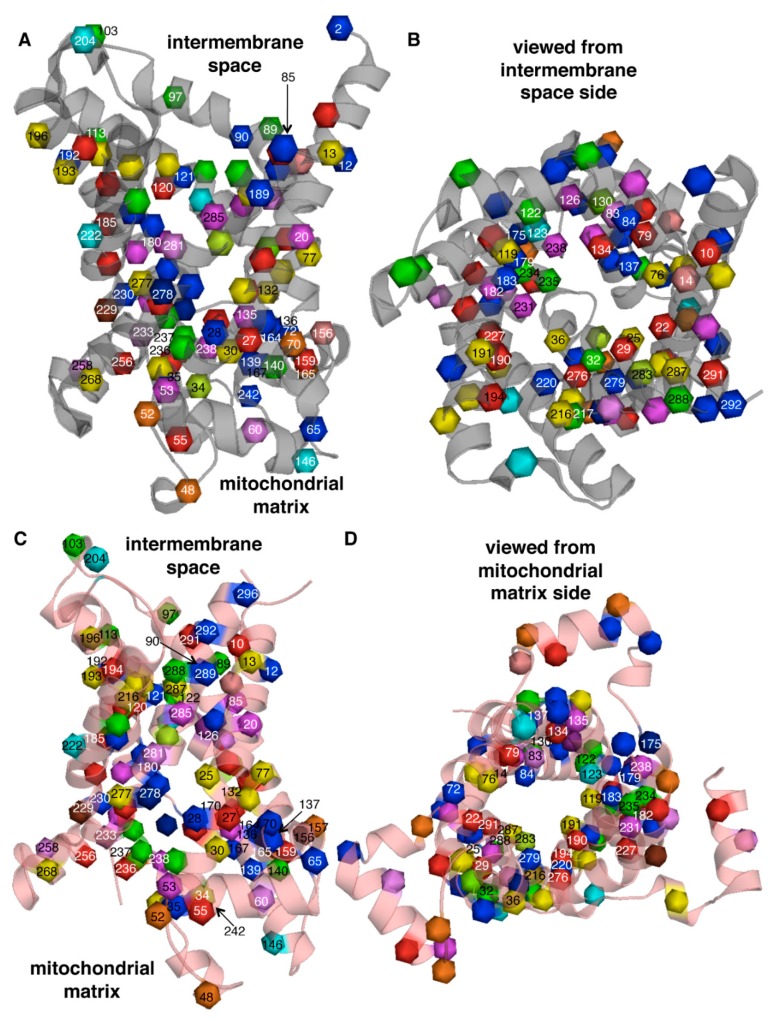
Structural positions of disease-causing point mutations in mitochondrial carriers. The bovine AAC1 structure (in the c-state, gray) and the thermophile AAC2 structure (in the m-state, pink) are viewed from the membrane plane (**A**,**C**) and the intermembrane space side (**B**,**D**). In all the panels, the α-carbons of the mutated positions are indicated as spheres colored as the top color of the mutations in the same position shown in the alignment of Figure 1. The numbering is that of bovine AAC1 (Figure 1) with the residue side chains exposed to the substrate translocation pore in B and D or outside the pore in A and C.

Most of the disease-causing mutations in MCs are single nucleotide substitutions (over 200). However, also insertions, deletions, and insertion/deletions (more than 70) of a varying number of nucleotides have been found (Table S1). It is possible to map the 166 disease-causing single nucleotide missense mutations (Figure 1) onto 100 different positions in the structures of AAC (Figure 2) due to the specific conserved sequence features of MCs, which makes structural alignments very reliable. Only 15% of these mutations are conservative mutations, i.e., substitutions with residues with similar chemical properties. Quite a large portion of the single-nucleotide missense mutations is found in the conserved residues of the SMSs (16%), in the contact point residues (10%), and 45% in the substrate translocation pore (Figure 2B,D). Of all the mutations, 25% are in lipid-exposed positions, 10% in buried positions within the protein, and the remaining 20% in mainly solvent-exposed positions (Figure 2A,C with all the positions outside the substrate translocation pore). Not surprisingly, these numbers suggest that the point mutations in the substrate translocation pore are the most common pathogenic missense mutations. Considering that only about 20% of the MC residues face the inside of the pore, 63% of these residue positions have at least one disease-causing point mutation. The corresponding numbers for the buried, lipid bilayer- and solvent-exposed positions are 29%, 30%, and 18%, respectively. Thus, it is clear that the substrate translocation pore residues are hotspots for disease-causing mutations.

All the diseases and respective phenotypes (23 until now), that have been associated to 18 MCs, are described below following the order of the SLC25 numbers.

### 3.2. SLC25A1 (Citrate Carrier, CIC) Deficiency

Since 2013 until now, 24 mutations in *SLC25A1* have been found in 27 patients suffering from combined D-2- and L-2-hydroxyglutaric aciduria [39,40,41,42,43,44,45] (Table 1 and Table S1). SLC25A1 deficiency patients exhibit lower levels of citrate and isocitrate in the urine as compared to controls. In contrast, the urinary excretion of the tricarboxylic acid (TCA) cycle intermediates 2-oxoglutarate, succinate, fumarate, and malate, besides that of D/L-2-hydroxyglutaric acids, is higher. SLC25A1 deficiency is generally characterized by a severe neurodevelopmental phenotype, including neonatal encephalopathy, respiratory insufficiency, developmental delay, hypotonia, seizures, and early death. Ventriculomegaly, congenital heart defects, lactic acidosis and mitochondrial respiratory chain deficiency have also been described in some patients. The genetic trait of the disease follows a recessive pattern of inheritance with patients presenting alleles with combinations of missense, nonsense, and frameshift mutations. Moreover, two mutations (p.Asp69Tyr and p.Arg247Gln) causing SLC25A1 deficiency are associated with congenital myasthenic syndromes and mild intellectual disability [41]. In addition, few patients manifested combined D-2- and L-2-hydroxyglutaric aciduria and diGeorge syndrome caused by a *SLC25A1* mutation on one allele and a microdeletion of 22q11.2, which contains *SLC25A1* and two other genes, on the homologous chromosome [45].

**Figure 3:**
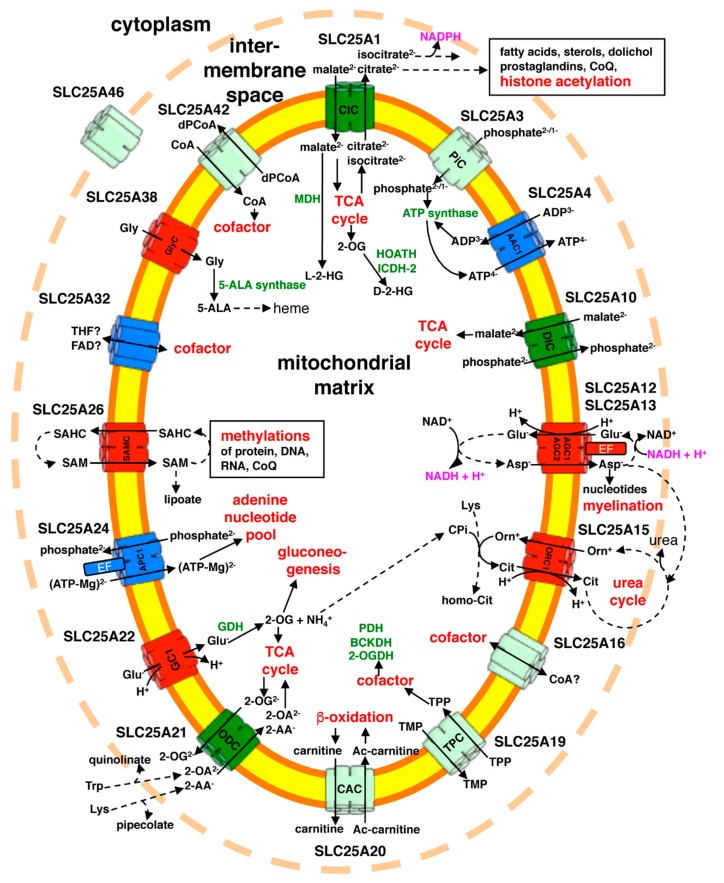
Metabolic roles of the mitochondrial carriers associated with diseases. When known, the substrate species transported by the carriers are shown. The carriers for carboxylates, amino acids, and nucleotides are colored in green, red, and blue, respectively, whereas the remaining MCs are colored in light lime. Enzymes are abbreviated in green. Other abbreviations are: 2-OA, 2-oxoacid; 2-AA, 2-aminoadipate; 2-OA, 2-oxoadipate; 2-OG, 2-oxoglutarate; 2-OGDH, 2-oxoglutarate dehydrogease; 5-ALA, 5-aminolevulinic acid; Ac, acyl; BCKDH, branched chain ketoacid dehydrogenase; CPi, carbamoylphosphate; D-2HG, D-2-hydroxyglutarate; dPCoA, dephospho-CoA; EF, EF hand Ca2+-binding domains; GDH, glutamate dehydrogenase; HOATH, hydroxy-oxoacid transhydrogenase; ICDH-2, isocitrate dehydrogenase 2; L-2-HG, L-2-hydroxyglutarate; MDH, malate dehydrogenase; PDH, pyruvate dehydrogenase; SAHC, S-adenosylhomocysteine; SAM, S-adenosylmethionine; TCA, tricarboxylic acid; THF, tetrahydrofolate; TMP, thiamine monophosphate; TPP, thiamine pyrophosphate.

On the basis of its substrate specificity and other transport measurements, it has been suggested that the *SLC25A1* gene product CIC catalyzes the electroneutral exchange of cytoplasmic malate^2−^ for mitochondrial citrate^2−^ or isocitrate^2−^ formed in the TCA cycle (Figure 3) [46,47,48,49]. In the cytoplasm citrate is important in the regulation of glycolysis through a feedback mechanism and in the production of acetyl-CoA which is needed for the synthesis of fatty acids, sterols, prostaglandins, dolichol and coenzyme Q (CoQ), and for histone acetylation. The CIC-catalyzed export of mitochondrial isocitrate plays a role in a shuttle that supplies cytosolic NADPH [49]. These transport functions are of central importance in most cell types, as CIC expression has been detected in a wide range of tissues with the highest mRNA levels in the liver, kidney, pancreas, and heart [50].

Several approaches have been used to assess the effects of missense mutations associated with SLC25A1 deficiency on CIC activity. The *S. cerevisiae* homologue of CIC, Ctp1p, harboring three corresponding human disease-causing mutations was reconstituted in liposomes for transport assays with radioactive citrate [39,41]. Another method utilized isotope-labeled glucose fed to fibroblasts from seven patients or to fibroblasts deficient of CIC and containing plasmids expressing 17 disease-causing point mutations, and the cellular efflux of isotope-labeled citrate formed was quantified [40,51]. A third approach employed mutant versions of human CIC expressed in *Lactococcus lactis*, and their transport activities in plasma membrane-fused liposomes were measured with radioactive citrate [52]. These studies show that the mutations causing D-2- and L-2-hydroxyglutaric aciduria affect citrate transport activity of CIC to various degrees, and that, with a few exceptions, the relative transport activities obtained by the different approaches for a specific CIC mutant are similar. The importance of the position of the mutations in the CIC structural homology model is generally in agreement with their relevance for the CIC activity: mutations in the vicinity of the substrate binding site and in the SMS residues lining the substrate translocation pore (Figure 2B,D, at positions 29, 79, 134, 190, and 276) almost deplete transport activity, whereas other mutations localized in the pore of the protein (Figure 2, at positions 10, 22, 194, and 291) or outside the pore retain partial activity (Figure 2A,C, at positions 27, 120, 159, 185, 236, and 256). Pop et al. (2018) have suggested correlations between the measured activity of single CIC mutants and the functional/structural importance of the original residue, as evaluated by analyses of the strength of the evolutionary selection in that amino acid position [53], as well as between the calculated average CIC activity of the two-allele mutations found in the patients and the severity of the disease [51]. Furthermore, zebrafish has been used as a model for exploring the morphological effects of CIC dysfunction; knockout of *SLC25A1* causes mitochondrial loss, a decrease in brain size, and cardiac dysfunction [54], whereas the expression of mild, moderate, and severe CIC mutants in the knockout animal shows progressive morphological abnormalities and defective neuromuscular junction formation [41].

The connections between reduced CIC activity and the clinical symptoms of combined D-2- and L-2-hydroxyglutaric aciduria are not totally clear. It is likely that the reduced activity of the CIC mutants reduces citrate and isocitrate efflux from mitochondria, and, subsequently, from cells, which in turn leads to reduced urine levels of these metabolites. In contrast, the increased mitochondrial citrate and isocitrate levels, due to their diminished export, and the loss of glycolysis feedback inhibition by cytosolic citrate could increase the concentrations of other TCA cycle intermediates which may be exported from the mitochondria by other MCs and eventually diffuse into the urine. The accumulation of mitochondrial 2-oxoglutarate is partly converted to D-2-hydroxyglutarate by the action of hydroxy-oxoacid transhydrogenase or isocitrate dehydrogenase 2, and that of malate into L-2-hydroxyglutarate by malate dehydrogenase (Figure 3). It is known that mutations in other enzymes (L-2-hydroxyglutarate dehydrogenase, D-2-hydroxyglutarate dehydrogenase and isocitrate dehydrogenase 2) cause L-2-hydroxyglutaric aciduria or D-2-hydroxyglutaric acidura type 1 or type 2 [55] and not a combined excretion of these two hydroxyacids. All three of these diseases manifest neurodevelopmental symptoms reminiscent of those found in combined D-2- and L-2-hydroxyglutaric aciduria. High levels of D/L-2-hydroxyglutarate have been suggested to inhibit 2-oxoglutarate-dependent enzymes, cause oxidative stress, and have neurotoxic effects [55]. Furthermore, it is likely that the reduced cytoplasmic acetyl-CoA concentration, leading to defective lipid anabolism and histone acetylation, contributes to the severe neurodevelopmental symptoms of combined D-2- and L-2-hydroxyglutaric aciduria.

### 3.3. SLC25A3 (Phosphate Carrier, PiC) Deficiency

The mitochondrial phosphate carrier PiC, encoded by *SLC25A3*, provides phosphate for oxidative phosphorylation and several other matrix processes as well as facilitates the transport of other substrates across the inner mitochondrial membrane by acting as a counter-substrate of other MCs (Figure 3). Due to the alternative splicing of exons III-A and III-B of *SLC25A3*, two isoforms of PiC are produced: isoform A, expressed in the heart and muscle, and isoform B that is expressed at much lower levels in all tissues [56,57,58]. PiC transports phosphate + H^+^ across the mitochondrial membrane. In particular, in studies with isolated rat-liver mitochondria, where virtually only isoform B is present, PiC was shown to transport both phosphate^1−^ and phosphate^2−^ in symport with an equivalent amount of H^+^ [59].

Four mutations in *SLC25A3* have been found in seven individuals with PiC deficiency from four different families [60,61,62] (Table 1). In six patients, two homozygous mutations were found in exon 3A affecting isoform A only: the point mutation p.Gly72Glu, which is located in the substrate translocation cavity at position 14 and may interfere with substrate binding (Figure 2B) [62], and a splice site substitution (Table S1). The seventh patient was compound heterozygous with two mutations affecting both isoform A and B: a deletion-insertion located in the matrix helix between H5 and H6, and a single amino acid substitution (p.Leu200Trp of PiC isoforms A and p.Leu199Trp of isoform B), which is located in position 156 on the matrix side (Figure 2A,C) and probably restricts conformational changes [62]. All the patients displayed hypertrophic cardiomyopathy, skeletal myopathy, and often lactic acidosis. In the severe cases, the affected patients did not survive the first year of life, whereas other patients overcame the pre- or neo-natal symptoms to develop almost normally but with skeletal myopathy and exercise intolerance. The physiological effects of not having functional heart PiC have been reproduced in mice with inducible cardiac-specific deletion of *SLC25A3* [63]. These mice developed a cardiac phenotype reminiscent of SLC25A3 deficiency and (see Section 3.4) the recessive forms of SLC25A4 deficiency, that both involve altered transport of substrates and/or products of oxidative phosphorylation. Isolated cardiac mitochondria from SLC25A3 knockout mice displayed a reduced ATP synthesis rate, although phosphate transport capacity was not completely abolished, as seen by measuring the uptake of radioactive phosphate. The latter observation may be explained by the fact that there are other MCs capable of transporting phosphate, such as the ATP-Mg^2+^/phosphate carriers (APCs), dicarboxylate carrier (DIC), and various uncoupling proteins (UCP2, UCP5, and UCP6) [19,64,65,66,67,68,69,70]. Furthermore, in SLC25A3 deficiency patients who have mutations only in PiC isoform A, also PiC isoform B may partly compensate for the defective phosphate transport. Until now, the transport activity of the disease-causing PiC mutations has not been investigated in reconstituted liposomes using the EPRA method.

### 3.4. SLC25A4 (ADP/ATP Carrier 1, AAC1) Deficiency

Mutations in the *SLC25A4* gene, encoding AAC1, one of the four human AACs [71], have been reported to cause three different diseases: adult-onset autosomal-dominant progressive external ophthalmoplegia 2 (AdPEO2) [72] and recessive and dominant early-onset mitochondrial myopathy/cardiomyopathy assigned as mitochondrial DNA depletion syndrome (MTDPS) type 12B [73,74,75,76] and 12A, respectively [77] (Table 1). AAC1 is expressed mainly in heart and skeletal muscle, where it is the predominant AAC isoform, and has the key role in cellular energy metabolism of exchanging cytosolic ADP^3−^ for matrix ATP^4−^ electrophoretically to provide the substrate and export the product of ATP synthase (Figure 3) [29]. All three diseases associated to *SLC25A4* mutations are characterized by multiple deletions in mitochondrial DNA as well as a reduced respiratory chain and oxidative phosphorylation capacity. AdPEO2 has usually an onset in early adulthood and displays numerous ragged-red fibers in muscle biopsy, ptosis, and progressive muscle weakness, especially of the external eye muscle [72]. Other forms of AdPEO are caused by mutations in subunits of the mitochondrial DNA helicase (AdPEO3), polymerase (AdPEO1 and AdPEO4), and ribonucleotide reductase (AdPEO5), suggesting that AAC1 also has a role in the mitochondrial replication that lies beneath the mitochondrial DNA deletions. How AAC1 malfunction is connected to mitochondrial replication is not known, but it has been suggested to be through the disequilibration of mitochondrial nucleotide pools or increased reactive oxygen species production [72]. The patients with MTDPS-12B (recessive) manifest muscle ragged-red fibers, mild myopathy with exercise intolerance, and lactic acidosis, but no ophthalmoplegia [73,74,75,76]. Recently, a new phenotype of recessive MTDPS-12B has been reported that, besides hypertrophic cardiomyopathy, lactic acidosis, and exercise intolerance, also displays elevated L-2-hydroxyglutarate urine levels and no mitochondrial DNA deletions [78]. It was speculated by the same authors that the increased L-2-hydroxyglutarate excretion may be a secondary effect of NADH accumulation in the mitochondrial matrix. The symptoms of the 12A variant of MTDPS (dominant) are more severe because hypotonia is apparent already after birth, in infanthood respiratory insufficiency, requiring mechanical ventilation, and poor motor development leads to death in many cases [77].

It is striking that AdPEO is caused by single point mutations located outside the central translocation pore (p.Ala90Asp, p.Leu98Pro, p.Asp104Gly, and p.Ala114Pro at positions 89, 97, 103, and 113, respectively, in Figure 2A,C) with the exception of p.Val289Met (Figure 2B, at position 288), which is a conservative mutation located in the pore between the substrate binding area and the residues of the c-gate. By contrast, MTDPS-12B is caused by the deletion, frame shift, and point mutations close to the binding site (p.Ala123Asp and p.Gln218Pro at positions 122 and 217 in Figure 2) or the m-gate (p.Leu141Phe and p.Arg236Pro at positions 140 and 235); and MTDPS-12A is caused by mutations in contact point I (p.Arg80His at position 79) or in translocation pore SMS residues (Figure 1, p.Lys33Gln and p.Arg235Gly in Figure 2 at positions 33 and 234, respectively). Transport measurements have shown that the AdPEO point mutations outside the translocation pore have 24–56% activity as compared to that of the wild-type, whereas MTDPS-12B mutations p.Ala123Asp and p.Arg236Pro virtually abolish activity and the MTDPS-12A mutations p.Lys33Gln, p.Arg80His, and p.Arg235Gly have 0%, 24%, and 3% activity, respectively [73,77,79]. It appears, therefore, that the severity of the late-onset dominant AdPEO with respect to both MTDPS-12B and MTDPS-12A (which are more severe) correlates with the type of mutations, their position within the carrier structure, and the measured residual transport activity. In contrast, such a correlation between MTDPS-12B and MTDPS-12A is not clear.

### 3.5. SLC25A10 (Dicarboxylate Carrier, DIC) Deficiency

One patient with a severe neurodegenerative disorder, characterized by epileptic encephalopathy, complex I deficiency, and mitochondrial DNA depletion in skeletal muscle was shown to possess mutations in the gene *SLC25A10* [80]. The heterozygous mutations of the patient (a silent and an intron mutation in one allele and a frame shift mutation in the other, Table S1) resulted in reduced RNA quantity and malfunctional splicing, leading to the absence of the dicarboxylate carrier DIC (encoded by *SLC25A10*). Mammalian DIC was shown to transport dicarboxylates such as malate and succinate as well as inorganic anions such as phosphate, sulfate, and thiosulfate by a strict electroneutral counter-exchange (Figure 3) [64,67,68,81,82,83,84]. As shown by transport experiments with isolated mitochondrial membranes from patient fibroblasts fused with liposomes, the malate/phosphate exchange was dramatically reduced [80]. It was also observed that the ratios of NADPH/NADP^+^ and GSH/GSSG were decreased. The connection between the transport function of DIC and the disease phenotype is not clear but its roles in the transport of reducing equivalents, anaplerotic Krebs cycle intermediates or components of sulfur metabolism could contribute to increased sensitivity to oxidative stress. Oxidative damage may lead to the loss of mitochondrial DNA and, subsequently, to the symptoms observed. It might be that, in some tissues, other MCs that transport certain DIC substrates partly compensate for the lack of SLC25A10 activity: PiC and APCs transport phosphate [19,58,85]; the oxoglutarate carrier transports malate and succinate [86,87,88,89]; UCP2 transports malate, phosphate, and sulfate [69]; and UCP5 and UCP6 transport malate, succinate, phosphate, sulfate, and thiosulfate [70].

### 3.6. SLC25A12 (Aspartate-Glutamate Carrier 1, AGC1, Aralar) Deficiency

Three children with arrested psychomotor development, hypotonia, seizures, global hypomyelination, and reduced levels of N-acetylaspartate in the cerebral hemispheres were found to have missense mutations in the gene *SLC25A12* of the aspartate-glutamate carrier AGC1 [90,91]. The two human AGC isoforms AGC1 and (see Section 3.7) AGC2 consist of two domains: an N-terminal soluble domain and a C-terminal MC catalytic domain [18]. Both proteins transport aspartate and glutamate by an obligatory electrophoretic exchange of (glutamate^−^ + H^+^)_out_ for aspartate^−^_in_, and are regulated by their N-terminal Ca^2+^-binding domains which protrude into the intermembrane space [18,92,93]. Their main physiological function is being key components of the malate-aspartate shuttle (together with the oxoglutarate carrier, SLC25A11, OGC) to catalyze the transfer of reducing equivalents of NADH from the cytosol to the mitochondrial matrix [49,94]. Another function of AGC1 and AGC2 is the mitochondrial export of aspartate that is a precursor for protein and nucleotide biosynthesis (Figure 3). In some tissues of AGC1 deficiency patients, the transfer of reducing equivalent of NADH may be maintained by AGC2 or the glycerol-3-phosphate shuttle, and the mitochondrial export of aspartate may be compensated, at least partly, by AGC2 or UCP2. In fact, the symptoms of SLC25A12 deficiency are mainly associated with alterations in the central nervous system and muscle, where AGC1 is the predominantly expressed AGC isoform [18].

The effects of the pathogenic mutations in *SLC25A12* have been studied in some detail. Skeletal muscle mitochondria from an AGC1 deficiency-affected child with the homozygous disease-causing mutation p.Gln590Arg (located in the substrate translocation pore at position 283, Figure 2B,D) displayed much reduced ATP production, and recombinant reconstituted protein harboring the mutation had no transport activity [90]. Two other patients, two siblings with the homozygous mutation p.Arg252Gln (found in the SMS at position 34, Figure 1 and Figure 2A,C, outside the pore) exhibited similar symptoms as the previous one; and the activity of the mutated protein was dramatically reduced but not completely abolished [91]. Recently, another disease-causing mutation, p.Thr444Ile (Figure 2B,D, at position 130), has been found in the substrate translocation pore (Table S1), but the transport activity of this AGC1 variant has not been investigated [95].

The link between defective AGC1 and hypomyelination is probably due to the protein contribution to mitochondrial export of aspartate, which is needed for the formation of N-acetylaspartate necessary for myelin biosynthesis. In fact, mice lacking AGC1 display growth retardation, an impaired central nervous system function, and reduced brain levels of N-acetyl-aspartate [96]. Moreover, AGC1 depletion in neurons (Neuro2A cells) and oligodendrocytes (precursor cells) inhibits proliferation and N-acetylaspartate synthesis [97,98]. It is noteworthy that (i) single nucleotide polymorphisms in AGC1 have been suggested to be associated with multi-factorial disorders, such as autism [99], and (ii) a mutation in AGC1 of Dutch shepherd dogs causes reduced transport activity of aspartate and glutamate in reconstituted liposomes as well as inflammatory myopathy [100]. The clinical conditions, psychomotor development, and myelination were markedly improved in an AGC1 deficiency child by introducing a ketogenic diet through the gradual increase of the ratio of fat to protein and carbohydrates [101]. This finding suggests a direction of future treatments of AGC1 deficiency.

### 3.7. SLC25A13 (Aspartate-Glutamate Carrier 2, AGC2, Citrin) Deficiency

The second human isoform of the aspartate-glutamate carrier AGC2 (SLC25A13) is expressed ubiquitously, especially in the liver. As AGC1, the aspartate-glutamate isoform 2 (AGC2) is an important component of the malate-aspartate shuttle which transfers reducing equivalents of NADH from the cytosol to the mitochondrial matrix and provides aspartate in the cytosol that is needed, among other things, for the urea cycle (Figure 3) [18,49,102]. In the *SLC25A13* gene, 117 different mutations causing AGC2 deficiency have been identified, and the patients, most of them from East and South Asia, are classified into two clinical phenotypes: neonatal intrahepatic cholestasis caused by citrin deficiency (NICCD, also classified as neonatal-onset citrullinemia type II) and adult-onset type II citrullinemia (CTLN2) (Table 1 and Table S1) [103,104]. In Japan, the observed prevalence of NICCD and CTLN2 is about 1:17 000 and 1:100 000–1:230 000, respectively [105]. The observed prevalence of NICCD is close to the calculated one based on heterozygous carrier frequency, which is about 1:65 in Japan and China [104,106]. In addition to transient intrahepatic cholestasis, NICCD patients present hepatomegaly, citrullinemia, ketotic hypoglycemia, aminoacidemias, hypoproteinemia, and growth retardation. Other dysfunctional liver-related symptoms may also occur, such as hepatitis, jaundice, reduction of coagulation factors and hemorrhagic diathesis. The NICCD symptoms are generally not severe and gradually disappear during the first years of life. NICCD is treated by a lactose-free and medium-chain-triglyceride diet supplemented with fat-soluble vitamins. Some of the NICCD subjects may suddenly develop CTLN2 as adults (usually between 20–50 years of age). CTLN2 patients have an aversion to carbohydrate-rich food and are unable to consume alcohol. In contrast, they have a liking for foods rich in protein and fat, that are energy sources less dependent on the malate-aspartate shuttle. As a matter of fact, alcohol and sugar intake as well as medication and surgery may provoke the symptoms. On top of citrullinemia, CTLN2 is characterized by fatty liver and repeated episodes of hyperammonemia, which lead to encephalopathy. CTLN2 is often associated with neuropsychiatric symptoms, such as nocturnal delirium, aggression, irritability, hyperactivity, delusions, disorientation, restlessness, drowsiness, loss of memory, flapping tremor, convulsive seizures, and coma. Treatments of CTLN2 consist of lactose-restricted low-carbohydrate diets supplemented with medium-chain-triglycerides [107]. Recently, from mice model studies, a combined supplement of ornithine with alanine or aspartate to reduce ammonium levels in blood has been suggested [108]. Liver transplantation is a resolutive remedy.

Among the many different AGC2 deficiency mutations identified, the pathogenic variant c.851-854del is the most common in Japan and China [105]. Besides the 68 more deleterious mutations (insertion, deletion, insertion/deletion, non-sense truncation, splicing, and frame shift mutations), 50 point mutations have been identified: 12 are found in the N-terminal soluble regulatory domain that harbours EF-hand Ca^2+^-binding motifs and 36 in the C-terminal mitochondrial carrier domain (Figure 1 and Table S1). Out of the latter group of mutations, fourteen are found in conserved motifs (Figure 1) and about half in the substrate translocation pore (Figure 2, at positions 29, 84, 130, 134, 137, 179, 182, 190, 220, 276, 279, 283, and 292).

### 3.8. SLC25A15 (Ornithine Carrier 1, ORC1) Deficiency

The ornithine carrier ORC1 is responsible for an important transport step in the urea cycle by catalyzing the electroneutral exchange of cytoplasmic ornithine^+^ for matrix citrulline + H^+^ (Figure 3) [49,102,109,110,111]. Mutations in the ORC1 gene, *SLC25A15*, have been identified to cause hyperornithinemia, hyperammonemia, and homocitrillinuria (HHH) syndrome in more than 100 patients, the majority of which lives in Canada, Italy, or Japan (Table 1) [112,113]. Besides the alterations underlined in the name of the disease, the metabolic features of HHH syndrome include elevated levels of polyamines, glutamine, and alanine as well as increased amounts of orotic acid and uracil in urine. Among the highly variable clinical symptoms of HHH syndrome are: in the acute phase, hepatitis-like attacks of vomiting, liver failure, confusion, and coma; and in the chronic phase lethargy, seizures, mental retardation, spastic paraplegia, cerebellar ataxia, learning difficulties, coagulation factor defects, and aversion to protein-rich food. Lethargy and coma are the most common early-onset symptoms, whereas movement and behaviour dysfunction are usually associated with late-onset or progressed stages of disease. Patients with HHH syndrome are treated in the acute phase with intravenous glucose, abolished protein intake, and ammonia scavengers such as benzoate and phenylbutyrate, and in the chronic phase with low-protein diets supplemented with citrulline or arginine. The development of HHH syndrome in patients under treatment is very variable from cases with only mild manifestations, compatible with an almost normal life, to cases with severe disability.

ORC1 deficiency reduces the rate of the urea cycle and hence leads to hyperammonemia, similarly to other genetic diseases associated with the urea cycle and ammonia-related metabolism (including AGC2 deficiency, Section 3.7). The ammonia toxicity is thought to be responsible for the neurological symptoms, which are in common to these diseases [114]. ORC1 deficiency also causes the build-up of ornithine in the cytosol and, subsequently, hyperornithinemia, increased levels of polyamines (which originate from cytosolic ornithine), and a secondary creatine deficiency, due to arginine-glycine amidotransferase inhibition by an excess of ornithine. Homocitrullinuria and increased excretion of orotic acid in urine are explained by an increase in carbamoyl-phosphate, which either condensates with lysine (forming homocitrulline) or enters the pyrimidine pathway (Figure 3).

Among the 38 different mutations found to cause HHH syndrome, the most common are p.Phe188del and p.Arg179*, which are found in 45% of the patients. The effects of many disease-causing mutations on ORC1-catalyzed transport have been assessed in reconstituted liposomes by the EPRA method and by in vitro cell culture studies, which have shown that, with very few exceptions, the mutations are deleterious for activity [111,115,116,117]. Some of the mutations (p.Glu180Lys, p.Arg275Gly and p.Arg275Gln at positions 183 and 279) (Figure 2B,D) are in the contact points (Figure 1), changing residues that were proved experimentally to participate directly in substrate binding [31]. Most point mutations are located along the substrate translocation pore (at positions 25, 36, 76, 119, 183, 191, 216, 220, 276, 279, and 287), but others have their residue side chains outside the pore of ORC1 (at positions 13, 28, 30, 35, 77, 132, 193, 196, 268, and 277).

### 3.9. SLC25A16 Deficiency

SLC25A16 was called Graves’ disease carrier protein because it was picked up in an immunoscreen with antisera from patients with Graves’ disease [118,119]. However, its association to Graves’ disease has never been clarified, although there are some clues about its function; SLC25A16 was shown to complement the lack of its yeast homologue Leu5p, which is implicated in mitochondrial CoA transport [120], and, furthermore, SLC25A16 is closely related to human SLC25A42, which was shown to transport CoA, dephospho-CoA, adenosine 3’,5’-diphosphate (PAP) and some other adenine nucleotides [121]. Recently, a mutation in SLC25A16 (p.Arg31Leu, located just before H1, Figure 1, at position 2 in Figure 2A) has been associated with autosomal recessive isolated fingernail dysplasia in members of a Pakistani family [122]. The affected members displayed a severe form of onychodystrophy, a hyperkertotic nail bed, a thickened and dystrophic nail plate, and digits with mild erythema and swelling close to the proximal nail folds, whereas the toenails were normal. The link between the nail malformation phenotype and the role of SLC25A16 in the mitochondrial CoA transport (if this is the role of this transporter) is yet unclear.

### 3.10. SLC25A19 (Thiamine Pyrophosphate Carrier, TPC) Deficiency

Patients suffering from the disorder Amish microcephaly have severe congenital microcephaly, 2-oxoglutaric aciduria, and often premature death. The traits of this disease were traced back in many generations of Amish families in Lancaster County (Pennsylvania) and linked to a mutation in *SLC25A19* causing the substitution p.Gly177Ala in its gene product [123]. Later, eight patients aged 3–20 years with less severe symptoms, such as increased lactate levels in cerebrospinal fluid or serum, chronic progressive polyneuropathy and fever-triggered acute episodes of flaccid paralysis and encephalopathy with bilateral striatal necrosis, were associated with five other mutations in *SLC25A19* [124,125,126,127]. This form of SLC25A19 deficiency was classified as thiamine metabolism dysfunction syndrome 4 (progressive polyneuropathy type).

*SLC25A19* encodes the thiamine pyrophosphate carrier TPC1 that transports thiamine pyrophosphate, thiamine monophosphate, and deoxynucleotides [123,128,129] as its homologue in *S. cerevisiae* Tpc1p [130]. The knockout of *SLC25A19* in the mouse causes depletion of mitochondrial thiamine pyrophosphate, embryonic lethality, brain malformations, and anemia [129]. It is therefore likely that the major symptoms of SLC25A19 deficiency are due to lack of mitochondrial import of thiamine pyrophosphate, which is formed in the cytoplasm from thiamine absorbed in the intestine, in exchange for intramitochondrial thiamine monophosphate (Figure 3). Mitochondrial thiamine pyrophosphate is an essential cofactor of three mitochondrial enzymes: pyruvate dehydrogenase, branched chain ketoacid dehydrogenase and 2-oxoglutarate dehydrogenase. The deficit of mitochondrial thiamine pyrophosphate causes reduced 2-oxoglutarate dehydrogenase activity, leading to the accumulation of 2-oxoglutarate and 2-oxoglutaric aciduria, and probably reduced pyruvate dehydrogenase activity, leading to the accumulation of lactate. Oral thiamine treatment has been reported to improve the conditions of some of the patients suffering from thiamine metabolism dysfunction syndrome 4 phenotype [125,127]. The severe phenotype-causing mutation p.Gly177Ala affects a highly conserved residue of the second part of the SMS (Figure 1, Figure 2B,D, at position 175); and the recombinant protein harboring this mutation was found to display a markedly reduced transport activity in isolated liposomes by the EPRA method [123,129]. In contrast, the mutations of the less severe phenotype may be less harmful to the activity because p.Gly125Ser and p.Gln192His (at positions 119 and 190, respectively), although located inside the substrate translocation pore, are not affecting conserved residues, and p.Ser194Pro and p.Leu290Gln (at positions 192 and 278, respectively) are outside the pore. However, p.Glu304Lys, which replaces a negative charge of the cytoplasmic gate (Figure 1, Figure 2B,C, at position 292) and therefore is expected to affect transport substantially, is also among the mutations causing the less severe phenotype. Unfortunately, the impact of the latter mutations on the transport function of SLC25A19 has not been investigated yet.

### 3.11. SLC25A20 (Carnitine-Acylcarnitine Carrier, CAC) Deficiency

The carnitine-acylcarnitine carrier CAC catalyzes the uptake of carnitine-linked long fatty acids into the mitochondria in exchange for free carnitine. This reaction is part of the carnitine cycle which allows the entry of fatty acids from the cytosol into the mitochondrial matrix where they are degraded by the β-oxidation pathway (Figure 3) [131,132]. Patients with CAC deficiency had been discovered before [133] the CAC gene (*SLC25A20*) was sequenced and the first disease-causing mutation was identified [35,134]. There are two clinical types of CAC deficiency: a neonatal-onset severe form and an infancy-onset milder form. Both phenotypes are characterized by lethargy, seizures, vomiting, fasting-induced coma, hypotonia, cardiomyopathy, muscle weakness, liver dysfunction and respiratory distress. Metabolic alterations include hypoglycemia, hypoketosis, hyperammonemia, dicarboxylic aciduria, increased long-chain acyl-carnitines, transaminases, and creatine kinase, whereas free carnitine is decreased. Fasting and illness may lead to brain damage, coma and ultimately death, even in the milder form. It is likely that the symptoms, with major effects on the liver, muscle, and brain, are due to accumulation of long-chain fatty acids and acyl-carnitines that cannot be oxidized or form ketone bodies. Similar symptoms are caused by mutations in the genes of β-oxidation enzymes. Mild form CAC deficiency patients usually respond well to a dietary treatment consisting of frequent, high-carbohydrate and low-fat meals supplemented with polysaturated fatty acids.

Only 12 out of the 38 CAC deficiency mutations are missense mutations, whereas the great majority are insertion, deletion, nonsense, frame shift, and splicing mutations (Table S1). Five of the CAC missense mutations are found in the substrate translocation pore (Figure 2B,D, at positions 25, 29, 79, 182, and 231), of which two are in the conserved SMS residues and two are in the contact point residues (Figure 1). The remaining missense mutations are outside the pore (Figure 2A,C, at positions 20, 53, 85, 135, 230, 238, and 285). Several approaches have been used to investigate the impact of the SLC25A20 mutations on the CAC transport. In the first method, indirect CAC activity was determined in fibroblasts by supplementing ^14^C-labeled fatty acids and measuring the rate of fatty acid oxidation as ^14^CO_2_ evolved/hr x mg cell protein [135]. All the other methods are based on the expression of the CAC variants in *Escherichia coli* [135,136] or in appropriate strains of *S. cerevisiae* [137] and *Aspergillus nidulans* [136,138].

### 3.12. SLC25A21 (Oxodicarboxylate Carrier, ODC) Deficiency

A homozygous missense mutation in *SLC25A21*, giving rise to the p.Lys232Arg replacement in the oxodicarboxylate carrier ODC, segregated with a patient with spinal muscular atrophy-like disease and mitochondrial myopathy associated with reduced mitochondrial DNA copy number (Table 1 and Table S1) [139]. ODC mainly transports 2-oxoadipate and 2-aminoadipate, that are produced from lysine and tryptophan degradation in the cytosol and are transported into the mitochondrial matrix, where they are oxidized and fed into the TCA cycle (Figure 3) [140,141]. It has been suggested by the same authors that the ODC-catalyzed mitochondrial import of these two metabolites occurs via an exchange for matrix 2-oxoglutarate [142]. Although the disease-causing p.Lys232Arg substitution is a conservative mutation, it affects an SMS residue (Figure 1) of the matrix salt bridge network (Figure 2B,D, at position 234), and renders SLC25A21 inactive [139]. In the SLC25A2-deficient patient, the urinary excretion of pipecolic and quinolinic acid, which are intermediates or by-products of the lysine and tryptophan degradation pathways, was observed together with that of 2-oxoadipate (Figure 3). The altered concentrations of these substances in the body are supposed to be toxic and lead to the reduction in mitochondrial DNA and cause mitochondrial dysfunction and spinal motor neuron abnormalities.

### 3.13. SLC25A22 (Glutamate Carrier 1, GC1) Deficiency

Initially, two mutations in *SLC25A22* were shown to cause severe neonatal epileptic encephalopathy with suppression bursts (NEESB) [143,144]. More recently, other eight mutations giving rise to similar symptoms have been identified in the same gene (all but one reviewed in [145]). This genetic disease is now classified as early infantile epileptic encephalopathy type 3 (EIEE3) (Table 1), and is characterized by early-onset myoclonic seizures, severe hypotonia, microcephaly, suppression burst pattern, severe developmental delay and often brain atrophy. A further mutation in *SLC25A22* caused hypotonia and developmental delay as in EIEE3, but also migrating partial seizures in infancy (MPSI) without suppression bursts [146]. Therefore, SLC25A22 deficiency patients present phenotypes spanning from very severe cases, with intractable epilepsy, no motor acquisition, vegetative state and early death, to moderate cases with tractable epilepsy, some motor acquisition, no microcephaly or suppression bursts [145].

*SLC25A22* encodes the glutamate carrier GC1, which imports glutamate by proton co-transport into the mitochondrial matrix [147] where it is transformed into ammonium ions and 2-oxoglutarate by glutamate dehydrogenase (Figure 3). The ammonium enters the urea cycle for clearance of excess nitrogen, whereas 2-oxoglutarate is used in gluconeogenesis (in liver and kidney) or oxidized in TCA cycle for ATP production (in non-hepatic tissues). SLC25A22 is highly expressed in most tissues including the brain, especially in areas associated with motor coordination and in astrocytes, that control the uptake of extracellular neurotransmitter glutamate [147,148]. It is likely that extracellular glutamate levels in brain are dysregulated in SLC25A22 deficiency patients [143] because it was shown that (i) reduced expression of SLC25A22 in glial cells leads to intracellular glutamate accumulation [148], and (ii) aberrant glutamate catabolism in astrocytes is associated with altered clearance of extracellular (synaptic) glutamate and early epileptic encephalopathy [149]. Moreover, some patients with SLC25A22 deficiency manifest hyperprolinemia [150], which might be explained by assuming that dysfunctional GC1 activity diminishes or abolishes the export of proline-derived glutamate from mitochondria leading to accumulation of proline in the body.

Out of ten point mutations in SLC25A22, two are in the first part of the third SMS, two in the same residue in contact point I (Figure 1), some are inside the substrate translocation pore (Figure 2, at positions 83, 119, and 220), and the rest outside the pore (at positions 60, 192, 230, 233, 258, and 281). It is noteworthy that patients with severe forms of SLC25A22 deficiency (severe EIEE3 and MPSI) have homozygous point mutations in residues with side chains protruding into the substrate translocation pore (with the exception of p.Pro206Leu), whereas milder forms exhibit the homozygous, or at least one of the heterozygous, mutations outside the pore. Moreover, GC1 variants of the most severe cases assessed in reconstituted liposomes by the EPRA method, exhibited negligible transport activity in vitro [143,144,146].

### 3.14. SLC25A24 (ATP-Mg^2+^/Phosphate Carrier 1, APC1) Deficiency

Gorlin–Chaudhry–Moss and Fontaine–Farriaux syndromes are rare dysmorphic genetic disorders manifested with similar symptoms: aged appearance with loose or wrinkled skin, short stature, hypertrichosis, skull deformities with craniosynostosis or brachycephaly, and a characteristic facial appearance of a depressed nasal bridge, low hairline, and microphthalmia [151]. Other common symptoms include digit and nail anomalies, cardiovascular abnormalities, umbilical hernia, and hypoplastic genital system. Whereas for some patients, early death has been reported, others display normal or nearly normal developmental outcomes. Given that both syndromes are caused by an autosomal dominant mutation in *SLC25A24*, either de novo p.Arg217Cys or p.Arg217His mutation [152,153], the two syndromes are now designated with a common name: Fontaine progeroid syndrome. Until now, 11 cases, which were confirmed to have one of the two above mentioned mutations, have been reported with this disease [151].

*SLC25A24* encodes the ATP-Mg^2+^/phosphate carrier APC1, which, besides the MC domain that transports ATP, ATP-Mg^2+^, ADP, AMP, and phosphate (Figure 3), has a regulatory N-terminal domain containing EF-hand Ca^2+^ binding sites activating transport when cytosolic Ca^2+^ increases [19,154,155]. This carrier is one of the four human isoforms and is involved in regulating the matrix adenine nucleotide pool through its capacity to transport adenine nucleotides in exchange for intra- or extra-mitochondrial phosphate [19]. The corresponding residue of APC1 Arg217 in the AAC structures is also an arginine located in a non-conserved position in the first part of the first SMS (Figure 1) on the matrix side of H1 (Figure 2A, at position 30). In the c-state, this arginine is buried in the structure and forms hydrogen bonds with residues of H2 and H3, while in the m-state it is positioned at the entrance of the substrate translocation pore and does not participate in any apparent interaction (Figure 2C). Therefore, it may be that the interactions of Arg217 play a role in the m-gate and neither its substitution with cysteine nor with histidine have the ability to form the same hydrogen bonds. Until now, the effects of the disease-causing mutations on the transport activity of APC1 are not known.

The connection between the mutations in *SLC25A24* and the symptoms of Fontaine progeroid syndrome is not clear. However, there are some clues: it was shown that fibroblasts from patients and cells expressing disease-causing mutant proteins have altered mitochondrial morphology, decreased proliferation rate, oxygen consumption, and mitochondrial ATP content, whereas mitochondrial membrane potential and oxidative stress sensitivity are increased [152,153]. These results may suggest that the above mentioned SLC25A24 mutations cause impaired energy metabolism, mitochondrial dysfunction, and a mitochondrial ATP deficit, which lead to the imbalanced proliferation and differentiation of progenitor cells involved in the development of skeletal and connective tissues [152,153]. Whether these consequences are due to APC1 haplo-insufficiency or active dominant effects of the mutated proteins remains to be answered.

### 3.15. SLC25A26 (S-Adenosylmethionine Carrier, SAMC) Deficiency

*SLC25A26* encodes mitochondrial S-adenosylmethionine carrier SAMC, which imports cytosol-synthesized S-adenosylmethionine (SAM) in exchange for matrix S-adenosylhomocysteine (Figure 3) [156], as its homologues in *S. cerevisiae* [157] and *A. thaliana* [158]. Matrix SAM is used as a methyl group donor in the methylation of DNA, RNA, proteins and coenzyme Q, and in the biosynthesis of lipoic acid. Therefore, it is not surprising that the three unrelated patients identified with SLC25A26 deficiency exhibited reduced intra-mitochondrial methylation of RNA and proteins, reduced translation and diminished coenzyme Q and lipoic acid levels [159]. The consequent mitochondrial dysfunction and defective respiratory chain activity may explain the clinical features found in patients suffering from this disorder, i.e., respiratory insufficiency, lactic acidosis, acute episodes of cardiopulmonary failure, and progressive muscle weakness.

The levels of transport activity of the recombinant SAMC variants harboring the three missense mutations associated with SLC25A26 deficiency seem to be related to their position in the SAMC homology model. p.Ala102Val and p.Pro199Leu, which cause almost depleted transport activity compared to the wild-type carrier protein and are found inside the substrate translocation pore (Figure 2, at position 126) and in the third SMS (at position 229, outside the pore), respectively, and p.Val148Gly, which causes a marked decrease in activity (15% of the wild-type protein), is found outside the pore (at position 180) [159]. Interestingly, the homozygous patient with the latter mutation displayed milder manifestations of the symptoms with respect to the heterozygous patient having the two first-mentioned mutations. Furthermore, the most severe case, which had a homozygous splice site mutation resulting in undetected protein product, died of respiratory and multiple organ failure in the first days of life. Therefore, the development and severity of the disease seem to be related to the transport activities of the mutated proteins and the allele composition of the patients.

### 3.16. SLC25A32 Deficiency

Two cases of SLC25A32 deficiency have been reported. One patient had recurrent late-onset exercise intolerance and abnormalities in the acyl-carnitine profile, which are characteristic features of multiple acyl-CoA dehydrogenase deficiency [160]. Later, another patient with more severe neuromuscular phenotype was identified presenting early onset ataxia, myoclonia, dysarthria, altered levels of organic acids and acylcarnithines in plasma and urine, muscle weakness, and exercise intolerance [161]. The state of both patients was improved upon treatment with riboflavin, which is a precursor of the cofactor FAD. The less severely affected patient had a truncation and a conservative p.Arg147His mutation (in the second positively charged residue of the second SMS (Figure 1) at position 139 outside the pore (Figure 2A,C)) in her two *SLC25A32* alleles, respectively; the more severe case had a homozygous oligonucleotide insertion/deletion mutation (Table S1), probably leading to the complete absence of the SLC25A32 protein. Until now, it has not been shown that SLC25A32 transports FAD and/or folate by direct transport assays. However, experiments have been performed suggesting that its *S. cerevisiae* homologue Flx1p (and also SLC25A32) transport FAD and/or folate (Figure 3) [162,163,164]. Furthermore, the missense mutation p.Arg147His of SLC25A32 introduced in a yeast Flx1 deletion strain complements the growth defect only partly. This may suggest that the mutant protein retains some activity [160] or the function of SLC25A32 is partially compensated by some other MC. It is known, for example, that the yeast NAD^+^ carrier transports FAD to some extent [165]. In addition, the physiological importance of SLC25A32 has been emphasized by gene-trap inactivation experiments of *SLC25A32* in mice displaying neural tube defects and embryonal death [166]. One might speculate that both reduced mitochondrial FAD and folate transports would lead to mitochondrial defects underlying the symptoms of malfunctioning SLC25A32.

### 3.17. SLC25A38 (Glycine Carrier, GlyC) Deficiency

Mutations in *SLC25A38* cause autosomal recessive nonsyndromic congenital sideroblastic anemia, which is characterized by ringed sideroblasts with accumulated iron deposits in mitochondria [167,168]. The onset of SLC25A38-associated sideroblastic anemia, which is usually microcytic, is within the first years of life. If untreated, toxic iron deposits build up in organs, such as heart and liver, causing cardiomyopathy and hepatic fibrosis, which lead to heart failure, chronic liver damage, and endocrine disorders, and subsequently can result in failure to thrive and death. The patients are treated with recurrent blood transfusions with the risk of increasing the iron overload even further. Therefore, much attention is focused on assessment and monitoring the iron levels. Thus, the transport function of SLC25A38 is associated with heme biosynthesis, and reconstituted GlyC (the protein product of *SLC25A38*) and its yeast homolog Hem25p were shown to transport glycine, which needs to enter the mitochondrial matrix to be converted into the heme precursor 5-aminolevulinic acid [169]. Mutations in mitochondrial 5-aminoluvelinate synthase 2, i.e., the enzyme catalyzing this reaction, also cause an X-linked form of nonsydromic congenital sideroblastic anemia. Out of the 25 pathogenic mutations found in *SLC25A38* so far, fifteen are single residue missense mutations (Figure 1 and Table S1). About half of the latter are found inside the substrate translocation pore (at positions 36, 119, 123, 191, 220, 276) and two precisely in the same residue of contact point II (at position 182, Figure 1 and Figure 2B,D).

### 3.18. SLC25A42 (CoA and PAP Carrier) Deficiency

A single nucleotide mutation in *SLC25A42* (causing the substitution p.Asn291Asp in its protein product) was found in 14 homozygous individuals of Arabic descent, who presented variable degrees of severity of symptoms such as mitochondrial myopathy with muscle weakness, lactic acidosis, encephalopathy, developmental regression, and epilepsy [170,171,172]. Recently, also a homozygous splice site mutation was found in a patient with similar clinical features [172]. As additional evidence of the pathogenic effects of certain *SLC25A42* mutations, a comparable phenotype with severe muscle disorganization and weakness was observed in the zebrafish knockdown of this gene [170]. *SLC25A42* encodes a MC that transports CoA, dephospho-CoA, adenosine 3’,5’-diphosphate (PAP), and some other adenine nucleotides [121]. The point mutation of Asn291 (Figure 2, at position 276) is localized inside the pore of the carrier structure in the vicinity of the substrate binding site and may therefore interfere with substrate binding. The main physiological function of SLC25A42 was suggested to be the import of CoA in exchange for PAP [121]. The possibility of external CoA and internal dephospho-CoA exchange was also considered [121]. In the mitochondrial matrix, CoA is an essential cofactor in many major processes, such as the TCA cycle, urea cycle, β-oxidation, heme biosynthesis, branched-chain amino acid oxidation, and protein acetylation. Moreover, in the mammalian mitochondrial matrix, PAP is formed when CoA donates its phosphopantetheine moiety to the acyl carrier protein for the synthesis of lipoic acid and long chain fatty acids. In *Drosophila melanogaster*, the main role of SLC25A42 has been suggested to be the export of mitochondrial dephospho-CoA [173]. At any rate, the disease-causing mutations affecting SLC25A42 transport activity probably give rise to a mitochondrial myopathy, which subsequently leads to the symptoms described above. Yet, it is difficult to comprehend the clinical heterogeneity and wide spectrum of severity of SLC25A42 deficiency even among patients with the same mutation in the same family.

### 3.19. SLC25A46 Deficiency

The clinical phenotypes resulting from mutations in *SLC25A46* are quite heterogeneous; they include axonal peripheral neuropathy, cerebellar, and optic atrophy found in patients diagnosed with congenital pontocerebellar hypoplasia, autosomal recessive cerebellar ataxia, Charcot–Marie–Tooth disease type 2 and optic atrophy spectrum disorder, Ramsay-Hunt or Leigh syndrome [174,175,176,177,178,179,180]. The severity and onset of the symptoms also vary, from death shortly after birth to mild manifestations appearing in adulthood. Unlike the other MCs, the SLC25A46 protein is localized in the outer mitochondrial membrane [174] and no transport activity has been associated with it so far. Instead, SLC25A46 has been suggested to play a role in mitochondrial fusion and fission because its overexpression and knockdown in cell lines lead to the fragmentation and hyperfusion of mitochondria, respectively [175]. In agreement to the symptoms observed in SLC25A46 deficiency patients, *SLC25A46* knockdown in zebrafish gives rise to defects in mitochondrial network maintenance, fission and cristae dynamics, and causes a dysfunctional neurodevelopment phenotype [174]. Notably, mutations in other genes involved in the regulation of the mitochondrial shape cause similar clinical features as those found in SLC24A46 deficiency patients [174]. Furthermore, it has been suggested that (i) the pathogenic SLC25A46 mutations affect the stability of the protein and interfere with protein–protein interactions important in mitochondrial morphology dynamics, and (ii) the degree of the protein destabilization and interference effects determine the severity of the disease [181]. Five of the pathogenic point mutations are located in different matrix helices (Figure 2A,C, at positions 48, 52, 70, 157, and 165) and they could be involved in protein-protein interactions; the other four are all in the SMS of H5 (Figure 1).

## 4. Multigenic Diseases with Mutations in MCs

There is clear evidence that mutations in MC genes cause the disorders described in Section 3. However, there are other diseases that are caused by mutations in several genes including MC genes or depend strongly on additional genetic and environmental factors. For example, it is noteworthy that polymorphisms in *SLC25A8* (encoding UCP2) and *SLC25A9* (UCP3) are associated with obesity and type 2 diabetes [182,183]; mutations in *SLC25A8* and other genes may lead to hyperinsulinism [184]; and a single nucleotide variation in *SLC25A40* (a carrier of unknown function) is linked to hypertriglyceridemia [185]. Furthermore, *SLC25A11* (encoding the oxoglutarate carrier OGC) is a tumor-suppressor gene (together with some genes encoding TCA cycle enzymes), i.e., mutations of *SLC25A11* confer a predisposition to metastatic paragangliomas [186]. In addition, genetic variants of several MCs have also been linked to complex multifactorial disorders such as autism [99,187,188].

## 5. Concluding Remarks

In the past decades, remarkable advances have been made in defining the molecular and genetic basis of different types of mitochondrial diseases, including those associated with MCs. Progress has been achieved despite the fact that many mitochondrial diseases are extremely heterogeneous clinically, biochemically, and genetically, and many of them are multi-system disorders affecting several organs [5]. As shown in this review, the number and understanding of the diseases associated with MCs is rapidly growing, and the connections between the mutations, defective transport, altered metabolism, and pathogenic mechanisms underlying the symptoms start to be better comprehended. This advance gives hope for improved, more rapid, and earlier diagnostics such as clinical observations, genetic counselling, prenatal diagnosis, and newborn screening, as well as for novel therapeutic strategies. However, this development is still hampered by the lack of knowledge about many MCs in terms of the substrates transported and their physiological roles in vivo in tissues and specialized cells. Future investigations are warranted along these lines of research to tackle and better comprehend the metabolic disorders associated with MCs.

Mitochondrial medicine aimed at correcting the mitochondrial defects is very challenging. Several complementary approaches, which include methods of general and personalized precision medicine, may be applied for efficient treatment. General approaches to improve and facilitate mitochondrial function utilize compounds such as antioxidants, factors that stimulate mitochondrial biogenesis (through signaling pathways and transcription factors), or clearance of defective mitochondria by autophagy and lysosomal removal [2,5]. For some of the MC-associated diseases, appropriate diets often supplemented with specific metabolites or cofactors are being used or are undergoing clinical trials. In principle, gene therapy is the resolutive approach that can cure the defects caused by specific gene alterations. Nowadays, viral vectors and/or CRISPR-Cas-based editing mutated DNA or RNA are available to accomplish gene therapy. These therapies aiming at correcting mutations in mtDNA are encountering many technical and physiological difficulties, such as a lack of mitochondrial vectors, multiple copies of mtDNA, and no systems for mtDNA recombination and repair. However, gene therapies for mitochondrial disorders caused by mutations in the nuclear DNA, such as the MC-associated diseases, may be developed in a relatively near future.

## Figures and Tables

**Table 1:** Diseases caused by mutations in mitochondrial carrier genes.

Affected MC	Phenotype	OMIM/Inheritance	Mutations/Patients	References of First Reported Case
SLC25A1, citrate carrier (CIC)	Combined D-2- and L-2-hydroxyglutaric aciduria Congenital myasthenic syndrome 23	615182/AR 618197/AR	24/40	(Edvardson et al., 2013) (Nota et al., 2013) (Chaouch et al., 2014)
SLC25A3, phosphate carrier (PiC)	Mitochondrial phosphate carrier deficiency	610773	4/7	(Mayr et al., 2007)
SLC25A4, ADP/ATP carrier 1 (AAC1)	Autosomal dominant progressive external ophthalmoplegia with mitochondrial DNA deletions 2 (AdPEO2) Mitochondrial DNA depletion syndrome (MTDPS) 12B (cardiomyopathic type) Mitochondrial DNA depletion syndrome (MTDPS) 12A (cardiomyopathic type)	609283/AD 615418/AR 617184/AD	5/9? 6/7 3/5	(Kaukonen et al., 2000) (Palmieri et al., 2005) (Thompson et al., 2016)
SLC25A10, dicarboxylate carrier (DIC)	Intractable epileptic encephalopathy with complex I deficiency	AR	3/1	(Punzi et al., 2018)
SLC25A12, aspartate/glutamate carrier 1 (AGC1)	Early infantile epileptic encephalopathy 39 (AGC1 deficiency)	612949/AR	3/4	(Wibom et al., 2009)
SLC25A13, aspartate/glutamate carrier 2 (AGC2)	Adult-onset citrullinemia type II (CTLN2) Neonatal-onset citrullinemia type II (NICCD)	603471/AR 605814/AR	117/>600	(Saheki and Kobayashi, 2002) (Kobayashi et al., 1999)
SLC25A15, ornithine carrier 1 (ORC1)	Hyperornithinemia-hyperammonemia-homocitrullinemia (HHH) syndrome	238970/AR	38/91	(Camacho et al., 1999)
SLC25A16	Fingernail dysplasia	AR	1/9	(Khan et al., 2018)
SLC25A19, thiamine pyrophosphate carrier (TPC)	Amish microcephaly Thiamine metabolism dysfunction syndrome 4 (progressive polyneuropathy type)	607196/AR 613710/AR	1/? 5/8	(Rosenberg et al., 2002) (Spiegel et al., 2009)
SLC25A20, carnitine/acylcarnitine carrier (CAC)	Carnitine-acylcarnitine translocase deficiency (CAC deficiency)	212138/AR	38/43?	(Huizing et al., 1997)
SLC25A21, oxodicarboxylate carrier (ODC)	Mitochondrial DNA depletion and spinal muscular atrophy–like disease	618811	1/1	(Boczonadi et al., 2018)
SLC25A22, glutamate carrier 1 (GC1)	Early infantile epileptic encephalopathy 3 (EIEE3)	609304/AR	11/17	(Molinari et al., 2005)
SLC25A24, ATP-Mg/phosphate carrier 1 (APC1)	Fontaine progeroid syndrome	612289/AD	2/11	(Ehmke et al., 2017) (Writzl et al., 2017)
SLC25A26, S-adenosylmethionine carrier (SAMC)	Combined oxidative phosphorylation deficiency 28	616794/AR	4/3	(Kishita et al., 2015)
SLC25A32	Riboflavin-responsive exercise intolerance	616839/AR	3/2	(Schiff et al., 2016)
SLC25A38, glycine carrier (GlyC)	Congenital sideroblastic anemia 2 (pyrodoxine-refractory)	205950/AR	25/39	(Guernsey et al., 2009)
SLC25A42, CoA and PAP carrier	Recurrent metabolic crises with variable encephalomyopathic features and neurologic regression	618416/AR	2/15	(Shamseldin et al., 2015)
SLC25A46	Hereditary motor and sensory neuropathy type VIB	616505/AR	16/25	(Abrams et al., 2015)

AD, autosomal dominant; AR autosomal recessive.

## Supplementary Materials

The following are available online at https://www.mdpi.com/2218-273X/10/4/655/s1, Table S1: Disease-causing mutations in mitochondrial carriers.

## Abbreviations

CoA: coenzyme A; CoQ, coenzyme Q; EPRA, expression-purification-reconstitution-transport-assay; MC, mitochondrial carrier; SAM, S-adenosylmethionine; SLC25, solute carrier family 25; SMS, signature motif sequence; TMP, thiamine monophosphate; TPP, thiamine pyrophosphate; and UCP, uncoupling protein.
