# Supplementary material for: Diseases Caused by Mutations in Mitochondrial Carrier Genes SLC25: A Review
Source: Biomolecules. 2020 Apr 23;10(4):655. doi: 10.3390/biom10040655 (PMC7226361; doi:10.3390/biom10040655)
Supplement: Supplementary file 1 [file biomolecules-10-00655-s001.pdf]

SUPPLEMENTARY MATERIAL TO:

Diseases caused by mutations in mitochondrial carrier genes *SLC25*: a review

Authors: Ferdinando Palmieri[1,*], Pasquale Scarcia[1] and Magnus Monné[1,2,*]

Affiliations: [1]Department of Biosciences, Biotechnologies and Biopharmaceutics, Laboratory of Biochemistry and Molecular Biology, University of Bari Aldo Moro, Via E. Orabona 4, 70125 Bari, Italy,

[2]Department of Sciences, University of Basilicata, Via Ateneo Lucano 10, 85100 Potenza, Italy

***Corresponding authors:** F. Palmieri (Department of Biosciences, Biotechnologies and Biopharmaceutics, University of Bari, Via E. Orabona 4, 70125 Bari, Italy, 0039-0805443323, ferdpalmieri@gmail.com) and M. Monné (Department of Sciences, University of Basilicata, Via Ateneo Lucano 10, 85100 Potenza, Italy, magnus.monne@unibas.it).

Table S1. Disease-causing mutations in mitochondrial carriers. For each gene/carrier the mutations are listed in the following order: first the nonsense, deletion/insertion and splicing mutations, and afterwards the missense mutations.

Mitochondrial carrier	Disease-associated mutation	Mutation effect in the protein (or problem in splicing)	Homo- / Hetero-zygous patients	References
**SLC25A1, CIC**	c.18_24dup	p.Ala9Profs*82	2/1	(1) (2)
	c.517_526del	p.Arg173Glyfs*2	1/1	(1) (3)
	c.648_655del	p.Met218Serfs*25	0/1	(3)
	c.768C > G	p.Tyr256*	0/1	(1)
**<u>Position in Fig. 2</u>**	c.821C > T	p.Ala274Ilefs*24	2/4	(1)
10	c.82G > A	p.Ala28Thr	0/1	(3)
22	c.119T > A	p.Ile40Asn	0/1	(3)
27	c.134C > T	p.Pro45Leu	0/2	(1)(2)
29	c.139G > A	p.Glu47Lys	1/0	(3)
55	c.205G > T	p.Asp69Tyr	3/0	(4)
79	c.278G > A	p.Gly93Asp	1/0	(3)
120	c.389G > A	p.Gly130Asp	0/1	(5)
134	c.430G > C	p.Glu144Gln	1/0	(1)
159	c.499G > A	p.Gly167Arg	0/1	(1)
185	c.578C > G	p.Ser193Trp	1/1	(1)(3)
190	c.593G > A	p.Arg198His	1/0	(6)
194	c.605T > C	p.Met202Thr	0/2	(1)
227	c.713A > G	p.Asn238Ser	1/0	(7)
236	c.740G > A	p.Arg247Gln	12/0	(4)(8)(9)
256	c.784T > C	p.Cys262Arg	1/0	(3)

276	c.844C > T	p.Arg282Cys	2/1	(1)
276	c.844C > G	p.Arg282Gly	1/0	(1)
276	c.845G > A	p.Arg282His	0/1	(5)
291	c.890A > G	p.Tyr297Cys	0/1	(1)
**SLC25A3, PiC**	c.158-9A > G	intron splice site	4/0	(10)
	c.886-898delins7	p.296_300Ins	0/1	(11)
**Position in Fig. 2**				
14	c.215G > A	p.Gly72Glu	2/0	(12)
156	c.599T > G	p.Leu200Trp	0/1	(11)
**SLC25A4, AAC1**	c.111+1G > A	p.Gln39Leufs*14	0/1	(13)[1]
	c.116_137del	no exon 1	1/0	(14)[1]
	c.390del	p.Phe130Leufs*41	0/1	(15)[1]
**Position in Fig. 2**				
32	c.97A > T	p.Lys33Gln	0/1	(16)[2]
79	c.239G > A	p.Arg80His	1/0	(17)[2]
89	c.269C > A	p.Ala90Asp	0/3	(18)[3]
97	c.293T > C	p.Leu98Pro	0/2	(19)[3]
103	c.311A > G	p.Asp104Gly	0/4	(20)[3]
113	c.340C > G	p.Ala114Pro	?	(21)[3]
122	c.368C > A	p.Ala123Asp	1/1	(15)[1] (22)[1]
140	c.423G > C	p.Leu141Phe	4/0	(15)[1]
217	c.653A > C	p.Gln218Pro	1/0	(23)
234	c.703C > G	p.Arg235Gly	3/0	(17)[2]
235	c.707G > C	p.Arg236Pro	0/1	(13)[1]
288	c.865G > A	p.Val289Met	?	(21)[3]
**SLC25A10, DIC**	c.304A >T	p.Lys102*	0/1	(24)
	c.790–37G > A[4]	intron	0/1	(24)
	c.684C > T[4]	p.Pro228Pro	0/1	(24)
**SLC25A12, AGC1**				
**Position in Fig. 2**				
34	c.1058G > A	p.Arg353Gln	2/0	(25)
130	c.1331C > T	p.Thr444Ile	1/0	(26)
283	c.1769A > G	p.Gln590Arg	1/0	(27)
**SLC25A13, AGC2**	c.-3251_c.15+18443del21709bp	gross deletion	?	(28)
	c.2T > C	missing start codon	0/3	(29)(30)(31)
	c.15G > A	splicing	0/8	(32)(33)
	g.16-2A > T	splicing	0/1	(34)
	r.16_212dup	Ex2_3dup	?	(35)
	c.46G > T	p.Glu16*	0/2	(32)
	c.70-862_212+3527del4532	deletion	0/1	(36)
	c.70_215del	deletion	0/1	(36)
	c.72T > A	p.Tyr24*	0/1	(34)
	c.127C > T	p.Arg43*	1/3	(37)
	c.172_173delGT	p.Val58Gfs*	2/0	(37)
	c.265delG	p.Asp89fs*	0/1	(34)

	IVS4ins6kb	p.Glu110fs*	0/7	(31)(34)
	c.329-1687_c.468+3865del	p.Glu110fs*	0/1	(31)(38)
	c.329-154_c468+252del2646;c468+2394_c468ins23	p.Glu110fs*	0/1	(31)
	c.448G > T	p.Glu150*	0/1	(34)
	c.475C > T	p.Gln159*	0/2?	(34)(39)
	c.478delC	p.Leu160Trpfs*	1/0	(40)
	c.493C > T	p.Gln165*	0/1	(31)
	c.495delA	p.Gln165*fs	0/1	(31)
	c.550C > T	p.Arg184*	0/7	(31)(32)(41)(42)(43)
	c.IVS6(1789bp)ins (c.615+1G > C)	p.Ala206fs*	1/1?	(32)
	c.615+1G > A	splicing	2/5?	(37)(39)(42)(43)(44)
	c.640C > T	p.Gln214*	0/1	(45)
	IVS6+5G > A (c.615+5G > A)	splicing	0/53	(31)(34)(41)(42)(43)(46)(47)(48)(49)
	c.650delT	p.Phe217fs*	?	(50)
	c.674C > A	p.Ser225*	8/19?	(33)(51)(52)(53)(54)(55)
	c.754G > A	p.Ala206fs*	0/3	(29)(34)(56)
	c.754+6T>G(IVS7+6T>G)	splicing?	?	(45)
	c.755-1G > C	p.252fs*	0/1	(31)
	c.755-2A > G	splicing	0/1	(57)
	c.775C > T	p.Gln259*	0/4	(31)(34)(42)(46)
	c.845_848+1delG	p.Gly283fs*	0/2	(31)(58)
	c.847G > T	p.Gly283*	0/1	(48)
	c.848+3A > C	splicing	0/1	(37)
	c.851_854del4 (c.852_855del4)	p.Arg284fs*	214/348?	(29)(30)(31)(33)(34)(37)(39)(42)(43)(45)(46)(47)(48)(51)(52)(53)(54)(59)(60)
	c.933G > A	splicing ?	0/1	(33)
	c.933_933ins+1GCAG	p.Ala312fs*	0/1	(31)
	c.955C > T	p.Arg319*	0/4	(34)(42)(47)(48)(57)
	c.985_986insT	p.Ala329fs*	0/1	(42)
	c.1019_1177del (c.1177+1G>A)	p.340_392del	74/102	(30)(31)(33)(34)(43)(48)(51)(52)(53)(54)(59)(61)(62)
	c.1019_c.1177+893del	p.340_392del	?	(63)
	c.1063C > T	p.Arg355*	0/3	(37)
	c.1078C > T	p.Arg360*	0/5?	(31)(32)(42)(43)(48)
	c.1092_1095delT	p.Phe365fs*	0/8	(31)(34)(41)(43)
	c.1146delA	p.Arg383fs*	0/1	(32)
	c.1189C > T	p.Gln397*	0/1	(32)
	c.1192_1193delT	p.Leu398fs*	?	(64)
	c.1231_1311del (c.1311+1G>A)	p.411_437del	5/26?	(33)(51)(53)(54)
	g.IVS13 + 2T >G	splicing	0/1	(32)
	c.1375delG	p.Ala459fs*	0/1	(32)
	c.1381G > T	p.Glu461*	0/1	(31)
	c.1399C > T	p.Arg467*	0/7?	(31)(34)(48)(64)
	c.1452+1G > A	splicing	0/1	(31)
	c.1453_1591dup	p.Met532fs*	4/0?	(32)(65)
	c.1453_1591del	p.Gly485fs*	0/1	(32)
	c.1610_1612del2ins2	p.Leu537Tyrfs*	2/0	(66)(67)
	c.1645C > T	p.Gln549*	0/1?	(34)(68)
	c.1638_1660dup23	p.Ala554Glyfs*	17/66?	(29)(31)(34)(37)(39)(42)(43)

				(45)(46)(47)(48)(51)(53)(54)
	Ex16+74_IVS17-32del516	p.Gln556fs*	2/6?	(32)(62)
	c.1706_1707delTA	p.Thr569fs*	0/1	(31)(34)
	c. 1709_1710insA(1706_1707insA)	p.Ile570fs*	?	(69)
	c.1736G > A	p.Trp579*	0/1	(34)
	IVS16ins3kb	p.Ala584fs*	7/109?	(29)(30)(31)(32)(34)(42)(43)(45)(46)(47)(48)
	c.1799_1800insA	p.Tyr600*	0/20?	(32)(33)(53)(54)
	c.1801G > T	p.Glu601*	0/15?	(32)(42)(52)(54)
	c.1813C > T	p.Arg605*	1/8?	(32)(53)(54)
	c.1841+3_1841+4delAA	splicing	0/2	(58)(60)
	c.74C > A	p.Ala25Glu	0/1	(37)
	c.103A > G	p.Met35Val	0/1	(31)
	c.115G > T	p.Asp39Tyr	?	(70)
	c.221C > T	p.Ser74Phe	?	(68)
	c.254T > C	p.Leu85Pro	1/0	(42)(43)
	c.284C > A	p.Ala95Asp	0/1	(45)
	c.287T > C	p.Phe96Ser	0/2	(42)
	c.415G > A	p.Gly139Arg	0/1	(34)
	c.443A > G	p.Tyr148Cys	0/1	(34)
	c.527G > T	p.Gly176Val	0/1	(34)
**Position in**	c.790G > A	p.Val264Ile	?	(71)
**Fig. 2**	c.869T > C	p.Ile290Thr	?	(50)
12	c.998G > A	p.Gly333Asp	0/1	(47)(48)
28	c.1046T > C	p.Ile349Thr	?	(72)
29	c.1048G > A	p.Asp350Asn	0/5	(31)(34)
34	c.1063C > G	p.Arg355Gly	0/2	(31)(34)
34	c.1064G > A	p.Arg355Gln	0/2	(31)(49)
35	c.1067T > A	p.Met356Lys	?	(72)
65	c.1157G > T	p.Gly386Val	0/1	(63)
72	c.1177G > A	p.Gly393Ser	0/1?	(31)(32)(39)
84	c.1215G > T	p.Lys405Asn	0/2	(34)
90	c.1231G > A	p.Val411Met	0/1	(48)
120	c.1307_1308del2ins2	p.Gly436Glu	0/1	(73)
121	c.1311C > T	p.Cys437Cys	1/0	(46)
130	c.1336A > C	p.Thr446Pro	0/1	(32)
134	c.1349A > G	p.Glu450Gly	0/1	(42)
136	c.1354G > A	p.Val452Ile	0/1	(74)
137	c.1357A > G	p.Lys453Glu	?	(75)
137	c.1358A > G	p.Lys453Arg	0/1	(64)
139	c.1364G > T	p.Arg455Leu	?	(34)
164	c.1420G > A	p.Val474Met	?	(76)
167	c.1430T > G	p.Leu477Arg	?	(77)
179	c.1465T > C	p.Cys489Arg	0/1	(32)(66)
182	c.1478A > G	p.Asp493Gly	0/2	(55)
190	c.1498T > G	p.Tyr500Asp	0/1	(34)
192	c.1505C > T	p.Pro502Leu NP	0/1	(46)(78)
220	c.1592G > A	p.Gly531Asp	0/1	(34)
230	c.1622C > A	p.Ala541Asp	0/11	(47)(48)(57)
235	c.1637C > T	p.Thr546Met	0/4	(34)
235	c.1637C > G	p.Thr546Arg	0/1	(37)
242	c.1658G > A	p.Arg553Gln	0/1	(39)
276	c.1754G > A	p.Arg585His	0/1?	(42)(43)
279	c.1763G > A	p.Arg588Gln	0/2	(32)
279	c.1763G > C	p.Arg588Pro	?	(68)

283	c.1775A > C	p.Gln592Pro	1/0?	(34)
289	c.1793T > G	p.Leu598Arg	0/1	(32)
292	c.1801G > A	p.Glu601Lys	2/0?	(32)(43)(54)
296	c.1814G > A	p.Arg605Gln NP	0/5	(29)(78)
	c.1895C > T	p.Pro632Leu NP	0/1	(32)(78)
	c.1915G > A	p.Gly639Ser	?	(77)
**SLC25A15, ORC1**	c.56+1G > T	intron/fs	1/0	(79)
	c.96_97insCA	p.Met33Glnfs*	2/0?	(80)(81)
	c.164insA	p.Tyr55*	0/1	(82)
	c.265C > T	p.Gln89*	1/0	(82)
	c.446delG	p.S149Tfs*	2/0	(83)
	c.525insC	p.Ser175fs*	0/1	(79)
	c.535C > T	p.Arg179*	9/2	(79)(81)(82)(84)(85)(86)
	c.553_564del	p.Phe188del	32/7?	(80)(81)(87)(88)(89)
	c.684_685insAAC	p.228_229insAsn	1/0	(84)
	c.733A > T	p.Lys245*	2/0	(79)
	g.823C > T	p.Arg275*	2/1	(90)(91)
	c.861insG	p.Ser290*	1/0	(82)
	13q14del	large del	0/1	(87)
	IVS5+1G > A	exon 5 skipping	1/0	(82)
**Position in Fig. 2**				
13	c.44C > A	p.Ala15Glu	1/0	(80)
13	c.44C > T	p.Ala15Val	1/0	(92)
25	c.79G > A	p.Gly27Arg	2/3	(79)(82)
25	c.80G > A	p.Gly27Glu	1/0	(84)
28	c.88T > G	p.Phe30Val	0/1	(93)
30	c.95C > G	p.Thr32Arg	5/0	(94)
35	c.110T > G	p.Met37Arg	1/0	(79)
36	c.113A > C	p.Gln38Pro	0/1	(93)
76	c.208_209delCAinsTT	p.Ala70Leu	2/0	(79)
77	c.212T > A	p.Leu71Gln	1/0	(79)
119	c.337G > T	p.Gly113Cys	0/1	(95)
132	c.377C > G	p.Pro126Arg	1/0	(96)
183	c.538G > A	p.Glu180Lys	0/1	(87)
191	c.564C > G	p.Phe188Leu	1/0	(79)
193	c.572G > A	p.Gly190Asp	0/1	(82)
196	c.568T > C	p.Leu193Pro	0/1	(89)
216	c.646G > A	p.Gly216Ser	1/0	(79)
220	c.658G > A	p.Gly220Arg	3/1	(91)(97)
268	c.790G > C	p.Ala264Pro	0/2	(81)
276	c.815C > T	p.Thr272Ile	1/2	(79)(86)
277	c.818T > A	p.Met273Lys	0/1	(95)
279	c.824G > A	p.Arg275Gln	1/0	(82)
279	?	p.Arg275Gly	0/2	?
287	c.847C > T	p.Leu283Phe	0/1	(79)
**SLC25A16** **Position in Fig. 2**				
2	c.92G > T	p.Arg31Leu	9/0	(98)
**SLC25A19, TPC** **Position in Fig. 2**				
119	c.373G > A	p.Gly125Ser	4/0	(99)
175	c.530G > C	p.Gly177Ala	?	(100)

190	c.576G > C	p.Gln192His	1/0	(101)
192	c.580T > C	p.Ser194Pro	1/0	(102)
278	c.869T > A	p.Leu290Gln	1/0	(103)
292	c.910G > A	p.Glu304Lys	1/0	(103)
**SLC25A20, CAC**	c.65_69insTGTGC	p.Leu24Cysfs*	0/1	(104)
	c.84delT	p.His29Thrfs*	0/2	(105)(106)
	c.106-2A>T	splicing	0/1	(107)
	c.160_163del4ins4	p.54_55delGT insWA	1/2	(108)(109)
	c.168delT	p.Phe56Leufs*	0/1	(108)
	c.180delG	p.Lys61Argfs*	1/0	(104)
	c.199 - 10T > G	splicing	6/4	(104)(105)(106)(107)(110)
	c.261_388del1128		0/1	(111)
	c.270delC	p.Phe91Leufs*	1/0	(105)
	c.326+1delG	splicing	1/2	(105)(112)(113)
	c.362delG	p.Gly121Alafs*	0/1	(109)
	c.496C > T	p.Arg166*	1/1	(104)(108)(114)
	c.528delT	p.Met177Cysfs*	0/1	(108)
	c.532C > T	p.Arg178*	1/3	(104)(109)(115)
	c.516T > A	p.Trp192*	0/2	(107)
	c.609 - 1G > A	splicing	1/2	(104)
	c.609-3C>G	splicing	0/1	(113)
	c.671_780del110		0/1	(111)
	c.718+1G>C	splicing	0/1	(109)
	c.752_761del10	p.Asp251Gfs*	0/1	(108)
	c.779_781delAAG	p.Glu260del	0/1	(108)
	c.804delG	p.Phe269Serfs*	1/1	(104)(108)
	c.823C > T	p.Arg275*	0/1	(108)
	c.843+4_843+50del47	splicing	1/0	(109)
	c.897insC	p.Asn300Glnfs*	1/0	(116)
**Position in Fig. 2**	26kbdel	Ex5-9del	0/1	(108)
20	c.67T > C	p.Cys23Arg	1/0	(108)
25	c.82G > T	p.Gly28Cys	1/0	(104)
29	c.94G > A	p.Asp32Asn	0/1	(104)
53	c.164C > T	p.Thr55Asn	0/1	(108)
79	c.241G > A	p.Gly81Arg	2 alleles?	(117)
85	c.260C > T	p.Ala87Val	0/1	(108)
135	c.397C > T	p.Arg133Trp	0/3	(108)(109)
182	c.533G > A	p.Arg178Gln	1/1	(104)
230	c.689C > G	p.Pro230Arg	0/1	(104)
231	c.691G > C	p.Asp231His	0/2	(104)(109)
238	c.713A > G	p.Gln238Arg	2/0	(118)(119)
285	c.842C > T	p.Ala281Val	1/0	(109)
**SLC25A21, ODC**				
**Position in Fig. 2**				
234	c.695A > G	p.Lys232Arg	1/0	(120)
**SLC25A22, GC1**	c.813_814delTG	p.Ala272Glnfs*	0/2	(121)
**Position in Fig. 2**				
60	c.166A > C	p.Thr56Pro	3/0	(122)
83	c.235G > A	p.Glu79Lys	0/1	(122)
83	c.235G > C	p.Glu79Gln	0/1	(123)
119	c.328G > C	p.Gly110Arg	2/0	(124)
192	c.617C > T	p.Pro206Leu	5/0	(125)(126)

220	c.706G > T	p.Gly236Trp	1/0	(127)
230	c.736T > C	p.Cys246Arg	0/1	(123)
233	c.746T > A	p.Val249Glu	0/1	(122)
258	c.818G > A	p.Arg273Lys	0/2	(121)
281	c.886G > A	p.Ala296Thr	2/0	(122)
**SLC25A24, APC1**				
**Position in Fig. 2**				
30	c.649C > T	p.Arg217Cys	0/2	(128)(129)
30	c.650G > A	p.Arg217His	0/9	(128)(129)(130)(131)
**SLC25A26, SAMC**	c.33+1G > A	splice site	1/0	(132)
**Position in Fig. 2**				
126	c.305C > T	p.Ala102Val	0/1	(132)
180	c.443T > G	p.Val148Gly	1/0	(132)
229	c.596C > T	p.Pro199Leu	0/1	(132)
**SLC25A32**	c.425G > A	p.Trp142*	0/1	(133)
**Position in Fig. 2**	c.-264_31delins14	missing start codon	1/0	(134)
139	c.440G > A	p.Arg147His	0/1	(133)
**SLC25A38**	c.175C > T	p.Gln59*	0/1	(135)
	c.324_325delCT	p.Tyr109Leufs*	2/5	(136)(137)
	c.324_330del7bp	p.Leu108fs*	1/0	(138)
	c.336_347del11bp	p.Lys112fs	0/1	(136)
	c.349C > T	p.Arg117*	7/2	(136)(137)
	c.790A > T	p.Lys264*	2/0	(136)
	IVS3-1G > A	splice site	1/0	(136)
	c.858delA	p.Ala286fs*	1/0	(138)
	c.879T > G	p.Tyr293*	0/1	(136)
**Position in Fig. 2**	c.912C > T	p.Arg305*	0/1	(136)
36	c.166C > A	p.Gln56Lys	1/0	(137)
77	c.281T > A	p.Ile94Asn	0/1	(139)
119	c.389G > A	p.Gly130Glu	0/1	(136)
123	c.400C > T	p.Arg134Cys	1/1	(135)(137)
123	c.401G > A	p.Arg134His	0/1	(136)
136	c.440T > A	p.Ile147Asn	1/0	(137)
146	c.469G > C	p.Gly157Arg	0/1	(139)
182	c.560G > C	p.Arg187Pro	0/3	(136)
182	c.560G > A	p.Arg187Gln	1/2	(135)(137)
185	c.569C > G	p.Pro190Arg	1/0	(137)
191	c.587T > C	p.Leu196Pro	1/0	(140)
204	c.625G > C	p.Asp209His	0/4	(136)(137)
220	c.683G > T	p.Gly228Val	4/0	(137)(138)
222	c.689T > C	p.Leu230Pro	1/0	(139)
276	c.832C > G	p.Arg278Gly	1/0	(137)
**SLC25A42, CoA and PAP carrier**	c.380 +2T > A	splice site	1/0	(141)
**Position in Fig. 2**				
276	c.871A > G	p.Asn291Asp	14/0	(141)(142)(143)

**SLC25A46**	1.897-KB DEL	missing start codon	2/0	(144)
	c.42 > G	p.Tyr14*	0/2	(145)
	c.165_166insC	p.His56fs*94	0/1	(146)
	c.283+3G > T	p.Ser32Thrfs*	1/0	(147)
	c.462+1G > A	intron	0/2	(145)
	c.736A > T	p.Arg246*	2/0	(145)
**Position in Fig. 2**	c.882_885dupTTAC	p.Asn296fs*297	0/1	(146)
48	c.413T > G	p.Leu138Arg	2/0	(148)
52	c.425C > T	p.Thr142Ile	1/0	(149)
70	c.479G > C	p.Trp160Ser	1/0	(150)
157	c.746G > A	p.Gly249Asp	0/1	(146)
165	c.770G > A	p.Arg257Gln	1/0	(151)
229	c.998C > T	p.Pro333Leu	0/1	(146)
231	c.1005A > T	p.Glu335Asp	3/0	(146)
236	c.1018C > T	p.Arg340Cys	6/0	(146)(150)(152)
237	c.1022T > C	p.Leu341Pro	2/0	(144)

1) Mitochondrial DNA depletion syndrome 12B (cardiomyopathic type); 2) Mitochondrial DNA depletion syndrome 12A (cardiomyopathic type); 3) Autosomal dominant progressive external ophthalmoplegia with mitochondrial DNA deletions 2; 4) Mutations found on the same allele; NP, non-pathogenic.

References

1. Nota, B., Struys, E. A., Pop, A., Jansen, E. E., Fernandez Ojeda, M. R., Kanhai, W. A., Kranendijk, M., van Dooren, S. J. M., Bevova, M. R., Sistermans, E. A., Nieuwint, A. W. M., Barth, M., Ben-Omran, T., Hoffmann, G. F., de Lonlay, P., McDonald, M. T., Meberg, A., Muntau, A. C., Nuoffer, J.-M., Parini, R., Read, M.-H., Renneberg, A., Santer, R., Strahleck, T., van Schaftingen, E., van der Knaap, M. S., Jakobs, C., and Salomons, G. S. (2013) Deficiency in SLC25A1, encoding the mitochondrial citrate carrier, causes combined D-2- and L-2-hydroxyglutaric aciduria. *Am. J. Hum. Genet.* **92**, 627–631

2. Prasun, P., Young, S., Salomons, G., Werneke, A., Jiang, Y.-H., Struys, E., Paige, M., Avantaggiati, M. L., and McDonald, M. (2015) Expanding the Clinical Spectrum of Mitochondrial Citrate Carrier (SLC25A1) Deficiency: Facial Dysmorphism in Siblings with Epileptic Encephalopathy and Combined D,L-2-Hydroxyglutaric Aciduria. *JIMD Rep.* **19**, 111–115

3. Pop, A., Williams, M., Struys, E. A., Monné, M., Jansen, E. E. W., De Grassi, A., Kanhai, W. A., Scarcia, P., Ojeda, M. R. F., Porcelli, V., van Dooren, S. J. M., Lennertz, P., Nota, B., Abdenur, J. E., Coman, D., Das, A. M., El-Gharbawy, A., Nuoffer, J.-M., Polic, B., Santer, R., Weinhold, N., Zuccarelli, B., Palmieri, F., Palmieri, L., and Salomons, G. S. (2018) An overview of combined D-2- and L-2-hydroxyglutaric aciduria: functional analysis of CIC variants. *J. Inherit. Metab. Dis.* **41**, 169–180

4. Al-Futaisi, A., Ahmad, F., Al-Kasbi, G., Al-Thihli, K., Koul, R., and Al-Maawali, A. (2020) Missense mutations in SLC25A1 are associated with congenital myasthenic syndrome type 23. *Clin. Genet.* **97**, 666–667

5. Edvardson, S., Porcelli, V., Jalas, C., Soiferman, D., Kellner, Y., Shaag, A., Korman, S. H., Pierri, C. L., Scarcia, P., Fraenkel, N. D., Segel, R., Schechter, A., Frumkin, A., Pines, O., Saada, A., Palmieri, L., and Elpeleg, O. (2013) Agenesis of corpus callosum and optic nerve hypoplasia due to mutations in SLC25A1 encoding the mitochondrial citrate transporter. *J. Med. Genet.* **50**, 240–245

6. Smith, A., McBride, S., Marcadier, J. L., Michaud, J., Al-Dirbashi, O. Y., Schwartzentruber, J., Beaulieu, C. L., Katz, S. L., FORGE Canada Consortium, Majewski, J., Bulman, D. E., Geraghty, M. T., Harper, M.-E., Chakraborty, P., and Lines, M. A. (2016) Severe Neonatal Presentation of Mitochondrial Citrate Carrier (SLC25A1) Deficiency. *JIMD Rep.* **30**, 73–79

7. Cohen, I., Staretz-Chacham, O., Wormser, O., Perez, Y., Saada, A., Kadir, R., and Birk, O. S. (2018) A novel homozygous SLC25A1 mutation with impaired mitochondrial complex V: Possible phenotypic expansion. *Am. J. Med. Genet. A*. **176**, 330–336

8. Chaouch, A., Porcelli, V., Cox, D., Edvardson, S., Scarcia, P., De Grassi, A., Pierri, C. L., Cossins, J., Laval, S. H., Griffin, H., Müller, J. S., Evangelista, T., Töpf, A., Abicht, A., Huebner, A., von der

Hagen, M., Bushby, K., Straub, V., Horvath, R., Elpeleg, O., Palace, J., Senderek, J., Beeson, D., Palmieri, L., and Lochmüller, H. (2014) Mutations in the Mitochondrial Citrate Carrier SLC25A1 are Associated with Impaired Neuromuscular Transmission. *J Neuromuscul Dis*. **1**, 75–90

9. Balaraju, S., Töpf, A., McMacken, G., Kumar, V. P., Pechmann, A., Roper, H., Vengalil, S., Polavarapu, K., Nashi, S., Mahajan, N. P., Barbosa, I. A., Deshpande, C., Taylor, R. W., Cossins, J., Beeson, D., Laurie, S., Kirschner, J., Horvath, R., McFarland, R., Nalini, A., and Lochmüller, H. (2020) Congenital myasthenic syndrome with mild intellectual disability caused by a recurrent SLC25A1 variant. *Eur. J. Hum. Genet.* **28**, 373–377

10. Mayr, J. A., Zimmermann, F. A., Horváth, R., Schneider, H.-C., Schoser, B., Holinski-Feder, E., Czermin, B., Freisinger, P., and Sperl, W. (2011) Deficiency of the mitochondrial phosphate carrier presenting as myopathy and cardiomyopathy in a family with three affected children. *Neuromuscul. Disord.* **21**, 803–808

11. Bhoj, E. J., Li, M., Ahrens-Nicklas, R., Pyle, L. C., Wang, J., Zhang, V. W., Clarke, C., Wong, L. J., Sondheimer, N., Ficicioglu, C., and Yudkoff, M. (2015) Pathologic Variants of the Mitochondrial Phosphate Carrier SLC25A3: Two New Patients and Expansion of the Cardiomyopathy/Skeletal Myopathy Phenotype With and Without Lactic Acidosis. *JIMD Rep.***19**, 59-66

12. Mayr, J. A., Merkel, O., Kohlwein, S. D., Gebhardt, B. R., Böhles, H., Fötschl, U., Koch, J., Jaksch, M., Lochmüller, H., Horváth, R., Freisinger, P., and Sperl, W. (2007) Mitochondrial phosphate-carrier deficiency: a novel disorder of oxidative phosphorylation. *Am. J. Hum. Genet.* **80**, 478–484

13. Körver-Keularts, I. M. L. W., de Visser, M., Bakker, H. D., Wanders, R. J. A., Vansenne, F., Scholte, H. R., Dorland, L., Nicolaes, G. a. F., Spaapen, L. M. J., Smeets, H. J. M., Hendrickx, A. T. M., and van den Bosch, B. J. C. (2015) Two Novel Mutations in the SLC25A4 Gene in a Patient with Mitochondrial Myopathy. *JIMD Rep*. **22**, 39–45

14. Echaniz-Laguna, A., Chassagne, M., Ceresuela, J., Rouvet, I., Padet, S., Acquaviva, C., Nataf, S., Vinzio, S., Bozon, D., and Mousson de Camaret, B. (2012) Complete loss of expression of the ANT1 gene causing cardiomyopathy and myopathy. *J Med Genet*. **49**, 146–50

15. Tosserams, A., Papadopoulos, C., Jardel, C., Lemière, I., Romero, N. B., De Lonlay, P., Wahbi, K., Voermans, N., Hogrel, J.-Y., and Laforêt, P. (2018) Two new cases of mitochondrial myopathy with exercise intolerance, hyperlactatemia and cardiomyopathy, caused by recessive SLC25A4 mutations. *Mitochondrion*. **39**, 26–29

16. King, M. S., Thompson, K., Hopton, S., He, L., Kunji, E. R. S., Taylor, R. W., and Ortiz-Gonzalez, X. R. (2018) Expanding the phenotype of de novo SLC25A4-linked mitochondrial disease to include mild myopathy. *Neurol Genet*. **4**, e256

17. Thompson, K., Majd, H., Dallabona, C., Reinson, K., King, M. S., Alston, C. L., He, L., Lodi, T., Jones, S. A., Fattal-Valevski, A., Fraenkel, N. D., Saada, A., Haham, A., Isohanni, P., Vara, R., Barbosa, I. A., Simpson, M. A., Deshpande, C., Puusepp, S., Bonnen, P. E., Rodenburg, R. J., Suomalainen, A., Õunap, K., Elpeleg, O., Ferrero, I., McFarland, R., Kunji, E. R. S., and Taylor, R. W. (2016) Recurrent De Novo Dominant Mutations in SLC25A4 Cause Severe Early-Onset Mitochondrial Disease and Loss of Mitochondrial DNA Copy Number. *Am. J. Hum. Genet.* **99**, 860–876

18. Deschauer, M., Hudson, G., Müller, T., Taylor, R. W., Chinnery, P. F., and Zierz, S. (2005) A novel ANT1 gene mutation with probable germline mosaicism in autosomal dominant progressive external ophthalmoplegia. *Neuromuscul. Disord.* **15**, 311–315

19. Napoli, L., Bordoni, A., Zeviani, M., Hadjigeorgiou, G. M., Sciacco, M., Tiranti, V., Terentiou, A., Moggio, M., Papadimitriou, A., Scarlato, G., and Comi, G. P. (2001) A novel missense adenine nucleotide translocator-1 gene mutation in a Greek adPEO family. *Neurology*. **57**, 2295–2298

20. Komaki, H., Fukazawa, T., Houzen, H., Yoshida, K., Nonaka, I., and Goto, Y. (2002) A novel D104G mutation in the adenine nucleotide translocator 1 gene in autosomal dominant progressive external ophthalmoplegia patients with mitochondrial DNA with multiple deletions. *Ann. Neurol.* **51**, 645–648

21. Kaukonen, J., Juselius, J. K., Tiranti, V., Kyttälä, A., Zeviani, M., Comi, G. P., Keränen, S., Peltonen, L., and Suomalainen, A. (2000) Role of adenine nucleotide translocator 1 in mtDNA maintenance. *Science*. **289**, 782–5

22. Palmieri, L., Alberio, S., Pisano, I., Lodi, T., Meznaric-Petrusa, M., Zidar, J., Santoro, A., Scarcia, P., Fontanesi, F., Lamantea, E., Ferrero, I., and Zeviani, M. (2005) Complete loss-of-function of the

heart/muscle-specific adenine nucleotide translocator is associated with mitochondrial myopathy and cardiomyopathy. *Hum. Mol. Genet.* **14**, 3079–3088

23. von Renesse, A., Morales-Gonzalez, S., Gill, E., Salomons, G. S., Stenzel, W., and Schuelke, M. (2019) Muscle Weakness, Cardiomyopathy, and L-2-Hydroxyglutaric Aciduria Associated with a Novel Recessive SLC25A4 Mutation. *JIMD Rep*. **43**, 27–35

24. Punzi, G., Porcelli, V., Ruggiu, M., Hossain, M. F., Menga, A., Scarcia, P., Castegna, A., Gorgoglione, R., Pierri, C. L., Laera, L., Lasorsa, F. M., Paradies, E., Pisano, I., Marobbio, C. M. T., Lamantea, E., Ghezzi, D., Tiranti, V., Giannattasio, S., Donati, M. A., Guerrini, R., Palmieri, L., Palmieri, F., and De Grassi, A. (2018) SLC25A10 biallelic mutations in intractable epileptic encephalopathy with complex I deficiency. *Hum. Mol. Genet.* **27**, 499–504

25. Falk, M. J., Li, D., Gai, X., McCormick, E., Place, E., Lasorsa, F. M., Otieno, F. G., Hou, C., Kim, C. E., Abdel-Magid, N., Vazquez, L., Mentch, F. D., Chiavacci, R., Liang, J., Liu, X., Jiang, H., Giannuzzi, G., Marsh, E. D., Yiran, G., Tian, L., Palmieri, F., and Hakonarson, H. (2014) AGC1 Deficiency Causes Infantile Epilepsy, Abnormal Myelination, and Reduced N-Acetylaspartate. *JIMD reports*. **14**, 77–85

26. Pfeiffer, B., Sen, K., Kaur, S., and Pappas, K. (2019) Expanding Phenotypic Spectrum of Cerebral Aspartate-Glutamate Carrier Isoform 1 (AGC1) Deficiency. *Neuropediatrics*. 10.1055/s-0039-3400976

27. Wibom, R., Lasorsa, F. M., Töhönen, V., Barbaro, M., Sterky, F. H., Kucinski, T., Naess, K., Jonsson, M., Pierri, C. L., Palmieri, F., and Wedell, A. (2009) AGC1 deficiency associated with global cerebral hypomyelination. *New England Journal of Medicine*. **361**, 489–495

28. Zhang, Z.-H., Lin, W.-X., Zheng, Q.-Q., Guo, L., and Song, Y.-Z. (2017) Molecular diagnosis of citrin deficiency in an infant with intrahepatic cholestasis: identification of a 21.7kb gross deletion that completely silences the transcriptional and translational expression of the affected SLC25A13 allele. *Oncotarget*. **8**, 87182–87193

29. Treepongkaruna, S., Jitraruch, S., Kodcharin, P., Charoenpipop, D., Suwannarat, P., Pienvichit, P., Kobayashi, K., and Wattanasirichaigoon, D. (2012) Neonatal intrahepatic cholestasis caused by citrin deficiency: prevalence and SLC25A13 mutations among Thai infants. *BMC Gastroenterol*. **12**, 141

30. Zhang, Z.-H., Lin, W.-X., Deng, M., Zhao, X.-J., and Song, Y.-Z. (2012) Molecular analysis of SLC25A13 gene in human peripheral blood lymphocytes: Marked transcript diversity, and the feasibility of cDNA cloning as a diagnostic tool for citrin deficiency. *Gene*. **511**, 227–234

31. Lin, W.-X., Zeng, H.-S., Zhang, Z.-H., Mao, M., Zheng, Q.-Q., Zhao, S.-T., Cheng, Y., Chen, F.-P., Wen, W.-R., and Song, Y.-Z. (2016) Molecular diagnosis of pediatric patients with citrin deficiency in China: SLC25A13 mutation spectrum and the geographic distribution. *Sci Rep*. **6**, 29732

32. Tabata, A., Sheng, J.-S., Ushikai, M., Song, Y.-Z., Gao, H.-Z., Lu, Y.-B., Okumura, F., Iijima, M., Mutoh, K., Kishida, S., Saheki, T., and Kobayashi, K. (2008) Identification of 13 novel mutations including a retrotransposal insertion in SLC25A13 gene and frequency of 30 mutations found in patients with citrin deficiency. *J. Hum. Genet.* **53**, 534–45

33. Komatsu, M., Yazaki, M., Tanaka, N., Sano, K., Hashimoto, E., Takei, Y., Song, Y.-Z., Tanaka, E., Kiyosawa, K., Saheki, T., Aoyama, T., and Kobayashi, K. (2008) Citrin deficiency as a cause of chronic liver disorder mimicking non-alcoholic fatty liver disease. *J. Hepatol.* **49**, 810–820

34. Song, Y.-Z., Zhang, Z.-H., Lin, W.-X., Zhao, X.-J., Deng, M., Ma, Y.-L., Guo, L., Chen, F.-P., Long, X.-L., He, X.-L., Sunada, Y., Soneda, S., Nakatomi, A., Dateki, S., Ngu, L.-H., Kobayashi, K., and Saheki, T. (2013) SLC25A13 gene analysis in citrin deficiency: sixteen novel mutations in East Asian patients, and the mutation distribution in a large pediatric cohort in China. *PLoS ONE*. **8**, e74544

35. Zhang, Z.-H., Lin, W.-X., Deng, M., Zhao, X.-J., and Song, Y.-Z. (2012) Molecular analysis of SLC25A13 gene in human peripheral blood lymphocytes: Marked transcript diversity, and the feasibility of cDNA cloning as a diagnostic tool for citrin deficiency. *Gene*. **511**, 227–234

36. Dimmock, D., Kobayashi, K., Iijima, M., Tabata, A., Wong, L.-J., Saheki, T., Lee, B., and Scaglia, F. (2007) Citrin deficiency: a novel cause of failure to thrive that responds to a high-protein, low-carbohydrate diet. *Pediatrics*. **119**, e773-777

37. Dimmock, D., Maranda, B., Dionisi-Vici, C., Wang, J., Kleppe, S., Fiermonte, G., Bai, R., Hainline, B., Hamosh, A., O'Brien, W. E., Scaglia, F., and Wong, L.-J. (2009) Citrin deficiency, a perplexing global disorder. *Mol. Genet. Metab.* **96**, 44–49

38. Zheng, Q.-Q., Zhang, Z.-H., Zeng, H.-S., Lin, W.-X., Yang, H.-W., Yin, Z.-N., and Song, Y.-Z. (2016) Identification of a Large SLC25A13 Deletion via Sophisticated Molecular Analyses Using Peripheral Blood Lymphocytes in an Infant with Neonatal Intrahepatic Cholestasis Caused by Citrin Deficiency (NICCD): A Clinical and Molecular Study. *Biomed Res Int*. **2016**, 4124263

39. Lin, J.-T., Hsiao, K.-J., Chen, C.-Y., Wu, C.-C., Lin, S.-J., Chou, Y.-Y., and Shiesh, S.-C. (2011) High resolution melting analysis for the detection of SLC25A13 gene mutations in Taiwan. *Clin. Chim. Acta*. **412**, 460–465

40. Köse, M. D., Kagnici, M., Özdemir, T. R., Erdur, C. B., Erdemir, G., Karakoyun, M., Guzin, Y., Ceylaner, S., and Genel, F. (2020) Clinical findings in five Turkish patients with citrin deficiency and identification of a novel mutation on SLC25A13. *J. Pediatr. Endocrinol. Metab.* **33**, 157–163

41. Lu, Y. B., Kobayashi, K., Ushikai, M., Tabata, A., Iijima, M., Li, M. X., Lei, L., Kawabe, K., Taura, S., Yang, Y., Liu, T.-T., Chiang, S.-H., Hsiao, K.-J., Lau, Y.-L., Tsui, L.-C., Lee, D. H., and Saheki, T. (2005) Frequency and distribution in East Asia of 12 mutations identified in the SLC25A13 gene of Japanese patients with citrin deficiency. *J. Hum. Genet.* **50**, 338–346

42. Chen, R., Wang, X.-H., Fu, H.-Y., Zhang, S.-R., Abudouxikuer, K., Saheki, T., and Wang, J.-S. (2013) Different regional distribution of SLC25A13 mutations in Chinese patients with neonatal intrahepatic cholestasis. *World J. Gastroenterol.* **19**, 4545–4551

43. Fu, H.-Y., Zhang, S.-R., Wang, X.-H., Saheki, T., Kobayashi, K., and Wang, J.-S. (2011) The mutation spectrum of the SLC25A13 gene in Chinese infants with intrahepatic cholestasis and aminoacidemia. *J. Gastroenterol.* **46**, 510–518

44. Saheki, T., Kobayashi, K., Iijima, M., Horiuchi, M., Begum, L., Jalil, M. A., Li, M. X., Lu, Y. B., Ushikai, M., Tabata, A., Moriyama, M., Hsiao, K.-J., and Yang, Y. (2004) Adult-onset type II citrullinemia and idiopathic neonatal hepatitis caused by citrin deficiency: involvement of the aspartate glutamate carrier for urea synthesis and maintenance of the urea cycle. *Mol. Genet. Metab.* **81 Suppl 1**, S20-26

45. Chong, S. C., Lo, P., Chow, C. W., Yuen, L., Chu, W. C. W., Leung, T. Y., Hui, J., and Scaglia, F. (2018) Molecular and clinical characterization of citrin deficiency in a cohort of Chinese patients in Hong Kong. *Mol Genet Metab Rep*. **17**, 3–8

46. Wen, P.-Q., Wang, G.-B., Chen, Z.-L., Cui, D., Yuan, Q., Song, P., Chen, S.-L., Liao, J.-X., and Li, C.-R. (2011) [SLC25A13 gene analysis in neonates with intrahepatic cholestasis caused by citrin deficiency]. *Zhongguo Dang Dai Er Ke Za Zhi*. **13**, 303–308

47. Song, Y.-Z., Li, B.-X., Chen, F.-P., Liu, S.-R., Sheng, J.-S., Ushikai, M., Zhang, C.-H., Zhang, T., Wang, Z.-N., Kobayashi, K., Saheki, T., and Zheng, X.-Y. (2009) Neonatal intrahepatic cholestasis caused by citrin deficiency: clinical and laboratory investigation of 13 subjects in mainland of China. *Dig Liver Dis*. **41**, 683–689

48. Song, Y.-Z., Deng, M., Chen, F.-P., Wen, F., Guo, L., Cao, S.-L., Gong, J., Xu, H., Jiang, G.-Y., Zhong, L., Kobayashi, K., Saheki, T., and Wang, Z.-N. (2011) Genotypic and phenotypic features of citrin deficiency: five-year experience in a Chinese pediatric center. *Int. J. Mol. Med.* **28**, 33–40

49. Zhang, Z.-H., Lin, W.-X., Deng, M., Zhao, S.-T., Zeng, H.-S., Chen, F.-P., and Song, Y.-Z. (2014) Clinical, molecular and functional investigation on an infant with neonatal intrahepatic cholestasis caused by citrin deficiency (NICCD). *PLoS ONE*. **9**, e89267

50. Bijarnia-Mahay, S., Häberle, J., Rüfenacht, V., Shigematsu, Y., Saxena, R., and Verma, I. C. (2015) Citrin deficiency: A treatable cause of acute psychosis in adults. *Neurol India*. **63**, 220–222

51. Kobayashi, K., Sinasac, D. S., Iijima, M., Boright, A. P., Begum, L., Lee, J. R., Yasuda, T., Ikeda, S., Hirano, R., Terazono, H., Crackower, M. A., Kondo, I., Tsui, L. C., Scherer, S. W., and Saheki, T. (1999) The gene mutated in adult-onset type II citrullinaemia encodes a putative mitochondrial carrier protein. *Nature Genetics*. **22**, 159–163

52. Tamamori, A., Okano, Y., Ozaki, H., Fujimoto, A., Kajiwara, M., Fukuda, K., Kobayashi, K., Saheki, T., Tagami, Y., and Yamano, T. (2002) Neonatal intrahepatic cholestasis caused by citrin deficiency: severe hepatic dysfunction in an infant requiring liver transplantation. *Eur. J. Pediatr.* **161**, 609–613

53. Yasuda, T., Yamaguchi, N., Kobayashi, K., Nishi, I., Horinouchi, H., Jalil, M. A., Li, M. X., Ushikai, M., Iijima, M., Kondo, I., and Saheki, T. (2000) Identification of two novel mutations in the SLC25A13 gene and detection of seven mutations in 102 patients with adult-onset type II citrullinemia. *Hum. Genet.* **107**, 537–545

54. Yamaguchi, N., Kobayashi, K., Yasuda, T., Nishi, I., Iijima, M., Nakagawa, M., Osame, M., Kondo, I., and Saheki, T. (2002) Screening of SLC25A13 mutations in early and late onset patients with citrin deficiency and in the Japanese population: Identification of two novel mutations and establishment of multiple DNA diagnosis methods for nine mutations. *Hum. Mutat.* **19**, 122–130

55. Takahashi, Y., Koyama, S., Tanaka, H., Arawaka, S., Wada, M., Kawanami, T., Haga, H., Watanabe, H., Toyota, K., Numakura, C., Hayasaka, K., and Kato, T. (2012) An elderly Japanese patient with adult-onset type II citrullinemia with a novel D493G mutation in the SLC25A13 gene. *Intern. Med.* **51**, 2131–2134

56. Lin, W.-X., Zhang, Z.-H., Deng, M., Cai, X.-R., and Song, Y.-Z. (2012) Multiple ovarian antral follicles in a preterm infant with neonatal intrahepatic cholestasis caused by citrin deficiency: a clinical, genetic and transcriptional analysis. *Gene*. **505**, 269–275

57. Song, Y., Sheng, J., Ushikai, M., Hwu, W., Zhang, C., and Kobayashi, K. (2008) [Identification and diagnosis of three novel mutations in SLC25A13 gene of neonatal intrahepatic cholestasis caused by citrin deficiency]. *Zhonghua Er Ke Za Zhi*. **46**, 411–415

58. Deng, M., Cheng, Y., Shu, S., Huang, Z., and Song, Y. (2019) [A novel SLC25A13 variant and the resultant aberrant transcript identified in a pedigree affected with citrin deficiency]. *Zhonghua Yi Xue Yi Chuan Xue Za Zhi*. **36**, 116–119

59. Ohura, T., Kobayashi, K., Tazawa, Y., Nishi, I., Abukawa, D., Sakamoto, O., Iinuma, K., and Saheki, T. (2001) Neonatal presentation of adult-onset type II citrullinemia. *Hum. Genet.* **108**, 87–90

60. Zhang, L., Li, Y., Shi, W., Gao, J., Tian, Y., Li, Y., Guo, Y., Cui, S., and Zhang, X. (2019) Identification of a novel splicing mutation in the SLC25A13 gene from a patient with NICCD: a case report. *BMC Pediatr*. **19**, 348

61. Tomomasa, T., Kobayashi, K., Kaneko, H., Shimura, H., Fukusato, T., Tabata, M., Inoue, Y., Ohwada, S., Kasahara, M., Morishita, Y., Kimura, M., Saheki, T., and Morikawa, A. (2001) Possible clinical and histologic manifestations of adult-onset type II citrullinemia in early infancy. *J. Pediatr.* **138**, 741–743

62. Takaya, J., Kobayashi, K., Ohashi, A., Ushikai, M., Tabata, A., Fujimoto, S., Yamato, F., Saheki, T., and Kobayashi, Y. (2005) Variant clinical courses of 2 patients with neonatal intrahepatic cholestasis who have a novel mutation of SLC25A13. *Metab. Clin. Exp.* **54**, 1615–1619

63. Zeng, H.-S., Lin, W.-X., Zhao, S.-T., Zhang, Z.-H., Yang, H.-W., Chen, F.-P., Song, Y.-Z., and Yin, Z.-N. (2016) SLC25A13 cDNA cloning analysis using peripheral blood lymphocytes facilitates the identification of a large deletion mutation: Molecular diagnosis of an infant with neonatal intrahepatic cholestasis caused by citrin deficiency. *Mol Med Rep*. **14**, 5189–5194

64. Xing, Y., Qiu, W., Ye, J., Han, L., Xu, S., Zhang, H., Gao, X., Wang, Y., and Gu, X. (2010) [Studies on the clinical manifestation and SLC25A13 gene mutation of Chinese patients with neonatal intrahepatic cholestasis caused by citrin deficiency]. *Zhonghua Yi Xue Yi Chuan Xue Za Zhi*. **27**, 180–185

65. Ben-Shalom, E., Kobayashi, K., Shaag, A., Yasuda, T., Gao, H.-Z., Saheki, T., Bachmann, C., and Elpeleg, O. (2002) Infantile citrullinemia caused by citrin deficiency with increased dibasic amino acids. *Mol. Genet. Metab.* **77**, 202–208

66. Hutchin, T., Preece, M. A., Hendriksz, C., Chakrapani, A., McClelland, V., Okumura, F., Song, Y.-Z., Iijima, M., Kobayashi, K., Saheki, T., McKiernan, P., and Baumann, U. (2009) Neonatal intrahepatic cholestasis caused by citrin deficiency (NICCD) as a cause of liver disease in infants in the UK. *J. Inherit. Metab. Dis.* **32 Suppl 1**, S151-155

67. Radha Rama Devi, A., and Naushad, S. M. (2018) SLC25A13 c.1610_1612delinsAT mutation in an Indian patient and literature review of 79 cases of citrin deficiency for genotype-phenotype associations. *Gene*. **668**, 190

68. Oh, S. H., Lee, B. H., Kim, G.-H., Choi, J.-H., Kim, K. M., and Yoo, H.-W. (2017) Biochemical and molecular characteristics of citrin deficiency in Korean children. *J. Hum. Genet.* **62**, 305–307

69. Wang, H., Shu, S., Chen, C., Huang, Z., and Wang, D. (2015) Novel mutations in the SLC25A13 gene in a patient with NICCD and severe manifestations. *J. Pediatr. Endocrinol. Metab.* **28**, 471–475

70. Bai, X. L., Zhang, Y. N., Yang, T. T., Pi, Y. L., and Dou, Z. Y. (2017) Clinical features of children with neonatal intrahepatic cholestasis caused by citrin deficiency and anaysis on SLC25A13 gene. *Maternal and Child Health Care of China*. **32**, 4748–4951

71. Zeng, H.-S., Zhao, S.-T., Deng, M., Zhang, Z.-H., Cai, X.-R., Chen, F.-P., and Song, Y.-Z. (2014) Inspissated bile syndrome in an infant with citrin deficiency and congenital anomalies of the biliary tract and esophagus: identification and pathogenicity analysis of a novel SLC25A13 mutation with incomplete penetrance. *Int. J. Mol. Med.* **34**, 1241–1248

72. Ricciuto, A., and Buhas, D. (2014) A novel citrin deficiency mutation in a cholestatic infant. *J. Pediatr. Gastroenterol. Nutr.* **59**, e52

73. Fiermonte, G., Parisi, G., Martinelli, D., De Leonardis, F., Torre, G., Pierri, C. L., Saccari, A., Lasorsa, F. M., Vozza, A., Palmieri, F., and Dionisi-Vici, C. (2011) A new Caucasian case of neonatal intrahepatic cholestasis caused by citrin deficiency (NICCD): a clinical, molecular, and functional study. *Mol. Genet. Metab.* **104**, 501–506

74. Şeker-Yılmaz, B., Kör, D., Tümgör, G., Ceylaner, S., and Önenli-Mungan, N. (2017) p.Val452Ile mutation of the SLC25A13 gene in a Turkish patient with citrin deficiency. *Turk. J. Pediatr.* **59**, 311–314

75. Xu, J., Gao, M., Lyu, Y., Tang, Y., Wei, X., Yang, L., Zhang, K., Liu, Y., and Gai, Z. (2018) [Analysis of SLC25A13 gene mutations in five infants with neonatal intrahepatic cholestasis caused by citrin deficiency]. *Zhonghua Yi Xue Yi Chuan Xue Za Zhi*. **35**, 34–38

76. Zhang, Z.-H., Yang, Z.-G., Chen, F.-P., Kikuchi, A., Liu, Z.-H., Kuang, L.-Z., Li, W.-M., Song, Y.-Z., Kure, S., and Saheki, T. (2014) Screening for five prevalent mutations of SLC25A13 gene in Guangdong, China: a molecular epidemiologic survey of citrin deficiency. *Tohoku J. Exp. Med.* **233**, 275–281

77. Xiaoli, X., Suqi, Y., Yan, D., Lishan, Z., Peng, C., and Dongchi, Z. (2015) Clinical research of neonatal intrahepatic cholestasis caused by Citrin deficiency in Hubei Province. *Journal of Applied Clinical Pediatrics*. **30**, 1064–1068

78. Wongkittichote, P., Tungpradabkul, S., Wattanasirichaigoon, D., and Jensen, L. T. (2013) Prediction of the functional effect of novel SLC25A13 variants using a S. cerevisiae model of AGC2 deficiency. *J. Inherit. Metab. Dis.* **36**, 821–830

79. Tessa, A., Fiermonte, G., Dionisi-Vici, C., Paradies, E., Baumgartner, M. R., Chien, Y.-H., Loguercio, C., de Baulny, H. O., Nassogne, M.-C., Schiff, M., Deodato, F., Parenti, G., Rutledge, S. L., Vilaseca, M. A., Melone, M. A. B., Scarano, G., Aldamiz-Echevarría, L., Besley, G., Walter, J., Martinez-Hernandez, E., Hernandez, J. M., Pierri, C. L., Palmieri, F., and Santorelli, F. M. (2009) Identification of novel mutations in the SLC25A15 gene in hyperornithinemia-hyperammonemia-homocitrullinuria (HHH) syndrome: a clinical, molecular, and functional study. *Human Mutation*. **30**, 741–748

80. Shih, V. E., and Ficicioglu, C. (2000) Genotype and phenotype findings in the hyperornithinemia-hyperammonemia-homocitrullinuria (HHH) syndrome. *Journal of Inherited Metabolic Diseases*. **23**, 72

81. Kim, S. Z., Song, W. J., Nyhan, W. L., Ficicioglu, C., Mandell, R., and Shih, V. E. (2012) Long-term follow-up of four patients affected by HHH syndrome. *Clin. Chim. Acta*. **413**, 1151–1155

82. Salvi, S., Dionisi-Vici, C., Bertini, E., Verardo, M., and Santorelli, F. M. (2001) Seven novel mutations in the ORNT1 gene (SLC25A15) in patients with hyperornithinemia, hyperammonemia, and homocitrullinuria syndrome. *Hum. Mutat.* **18**, 460

83. Korman, S. H., Kanazawa, N., Abu-Libdeh, B., Gutman, A., and Tsujino, S. (2004) Hyperornithinemia, hyperammonemia, and homocitrullinuria syndrome with evidence of mitochondrial dysfunction due to a novel SLC25A15 (ORNT1) gene mutation in a Palestinian family. *J. Neurol. Sci.* **218**, 53–58

84. Tsujino, S., Kanazawa, N., Ohashi, T., Eto, Y., Saito, T., Kira, J., and Yamada, T. (2000) Three novel mutations (G27E, insAAC, R179X) in the ORNT1 gene of Japanese patients with hyperornithinemia, hyperammonemia, and homocitrullinuria syndrome. *Annals of neurology*. **47**, 625–31

85. Miyamoto, T., Kanazawa, N., Kato, S., Kawakami, M., Inoue, Y., Kuhara, T., Inoue, T., Takeshita, K., and Tsujino, S. (2001) Diagnosis of Japanese patients with HHH syndrome by molecular genetic analysis: a common mutation, R179X. *J. Hum. Genet.* **46**, 260–2

86. Lee, H. H. C., Poon, K. H., Lai, C. K., Au, K. M., Siu, T. S., Lai, J. P. S., Mak, C. M., Yuen, Y. P., Lam, C. W., and Chan, A. Y. W. (2014) Hyperornithinaemia-hyperammonaemia-homocitrullinuria syndrome: a treatable genetic liver disease warranting urgent diagnosis. *Hong Kong Med J.* **20**, 63–66

87. Camacho, J. A., Obie, C., Biery, B., Goodman, B. K., Hu, C.-A., Almashanu, S., Steel, G., Casey, R., Lambert, M., Mitchell, G. A., and Valle, D. (1999) Hyperornithinaemia- syndrome is caused by mutations in a gene encoding a mitochondrial ornithine transporter. *Nat. Genet.* **22**, 151–158

88. Debray, F.-G., Lambert, M., Lemieux, B., Soucy, J. F., Drouin, R., Fenyves, D., Dubé, J., Maranda, B., Laframboise, R., and Mitchell, G. A. (2008) Phenotypic variability among patients with hyperornithinaemia-hyperammonaemia-homocitrullinuria syndrome homozygous for the delF188 mutation in SLC25A15. *J. Med. Genet.* **45**, 759–764

89. Filosto, M., Alberici, A., Tessa, A., Padovani, A., and Santorelli, F. M. (2013) Hyperornithinemia-hyperammonemia-homocitrullinuria (HHH) syndrome in adulthood: a rare recognizable condition. *Neurol. Sci.* **34**, 1699–1701

90. Torisu, H., Kira, R., Kanazawa, N., Takemoto, M., Sanefuji, M., Sakai, Y., Tsujino, S., and Hara, T. (2006) A novel R275X mutation of the SLC25A15 gene in a Japanese patient with the HHH syndrome. *Brain & development*. **28**, 332–5

91. Tezcan, K., Louie, K. T., Qu, Y., Velasquez, J., Zaldivar, F., Rioseco-Camacho, N., and Camacho, J. A. (2012) Adult-onset presentation of a hyperornithinemia-hyperammonemia-homocitrullinuria patient without prior history of neurological complications. *JIMD Rep*. **3**, 97–102

92. Ersoy Tunali, N., Marobbio, C. M. T., Tiryakioğlu, N. O., Punzi, G., Saygılı, S. K., Onal, H., and Palmieri, F. (2014) A novel mutation in the SLC25A15 gene in a Turkish patient with HHH syndrome: functional analysis of the mutant protein. *Mol. Genet. Metab.* **112**, 25–29

93. Qadri, S. K., Ting, T. W., Lim, J. S., and Jamuar, S. S. (2016) Milder Form of Urea Cycle Defect Revisited: Report and Review of Hyperornithinaemia-Hyperammonaemia-Homocitrullinuria (HHH) Syndrome Diagnosed in a Teenage Girl Presenting with Recurrent Encephalopathy. *Ann. Acad. Med. Singap.* **45**, 563–566

94. Camacho, J. a, Mardach, R., Rioseco-Camacho, N., Ruiz-Pesini, E., Derbeneva, O., Andrade, D., Zaldivar, F., Qu, Y., and Cederbaum, S. D. (2006) Clinical and functional characterization of a human ORNT1 mutation (T32R) in the hyperornithinemia-hyperammonemia-homocitrullinuria (HHH) syndrome. *Pediatr. Res.* **60**, 423–429

95. Fecarotta, S., Parenti, G., Vajro, P., Zuppaldi, A., Della Casa, R., Carbone, M. T., Correra, A., Torre, G., Riva, S., Dionisi-Vici, C., Santorelli, F. M., and Andria, G. (2006) HHH syndrome (hyperornithinaemia, hyperammonaemia, homocitrullinuria), with fulminant hepatitis-like presentation. *J. Inherit. Metab. Dis.* **29**, 186–189

96. Miyamoto, T., Kanazawa, N., Hayakawa, C., and Tsujino, S. (2002) A novel mutation, P126R, in a Japanese patient with HHH syndrome. *Pediatr. Neurrol.* **26**, 65–67

97. Al-Hassnan, Z. N., Rashed, M. S., Al-Dirbashi, O. Y., Patay, Z., Rahbeeni, Z., and Abu-Amero, K. K. (2008) Hyperornithinemia-hyperammonemia-homocitrullinuria syndrome with stroke-like imaging presentation: clinical, biochemical and molecular analysis. *J. Neurol. Sci.* **264**, 187–194

98. Khan, S., Ansar, M., Khan, A. K., Shah, K., Muhammad, N., Shahzad, S., Nickerson, D. A., Bamshad, M. J., Santos-Cortez, R. L. P., Leal, S. M., and Ahmad, W. (2018) A homozygous missense mutation in SLC25A16 associated with autosomal recessive isolated fingernail dysplasia in a Pakistani family. *Br. J. Dermatol.* **178**, 556–558

99. Spiegel, R., Shaag, A., Edvardson, S., Mandel, H., Stepensky, P., Shalev, S. A., Horovitz, Y., Pines, O., and Elpeleg, O. (2009) SLC25A19 mutation as a cause of neuropathy and bilateral striatal necrosis. *Ann. Neurol.* **66**, 419–424

100. Rosenberg, M. J., Agarwala, R., Bouffard, G., Davis, J., Fiermonte, G., Hilliard, M. S., Koch, T., Kalikin, L. M., Makalowska, I., Morton, D. H., Petty, E. M., Weber, J. L., Palmieri, F., Kelley, R. I., Schäffer, A. A., and Biesecker, L. G. (2002) Mutant deoxynucleotide carrier is associated with congenital microcephaly. *Nat. Genet.* **32**, 175–179

101. Bottega, R., Perrone, M. D., Vecchiato, K., Taddio, A., Sabui, S., Pecile, V., Said, H. M., and Faletra, F. (2019) Functional analysis of the third identified SLC25A19 mutation causative for the thiamine metabolism dysfunction syndrome 4. *J. Hum. Genet.* **64**, 1075–1081

102. Ortigoza-Escobar, J. D., Alfadhel, M., Molero-Luis, M., Darin, N., Spiegel, R., de Coo, I. F., Gerards, M., Taylor, R. W., Artuch, R., Nashabat, M., Rodríguez-Pombo, P., Tabarki, B., Pérez-Dueñas, B., and Thiamine Deficiency Study Group (2017) Thiamine deficiency in childhood with attention to genetic causes: Survival and outcome predictors. *Ann. Neurol.* **82**, 317–330

103. Gowda, V. K., Srinivasan, V. M., Jehta, K., and Bhat, M. D. (2019) Bilateral Striatal Necrosis with Polyneuropathy with a Novel SLC25A19 (Mitochondrial Thiamine Pyrophosphate Carrier OMIMI*606521) Mutation: Treatable Thiamine Metabolic Disorder-A Report of Two Indian Cases. *Neuropediatrics*. **50**, 313–317

104. Costa, C., Costa, J. M., Slama, A., Boutron, A., Vequaud, C., Legrand, A., and Brivet, M. (2003) Mutational spectrum and DNA-based prenatal diagnosis in carnitine-acylcarnitine translocase deficiency. *Mol. Genet. Metab.* **78**, 68–73

105. Hsu, B. Y., Iacobazzi, V., Wang, Z., Harvie, H., Chalmers, R. A., Saudubray, J. M., Palmieri, F., Ganguly, A., and Stanley, C. A. (2001) Aberrant mRNA splicing associated with coding region mutations in children with carnitine-acylcarnitine translocase deficiency. *Mol. Genet. Metab.* **74**, 248–255

106. Ogawa, A., Yamamoto, S., Kanazawa, M., Takayanagi, M., Hasegawa, S., and Kohno, Y. (2000) Identification of two novel mutations of the carnitine/acylcarnitine translocase (CACT) gene in a patient with CACT deficiency. *J. Hum. Genet.* **45**, 52–55

107. Fukushima, T., Kaneoka, H., Yasuno, T., Sasaguri, Y., Tokuyasu, T., Tokoro, K., Fukao, T., and Saito, T. (2013) Three novel mutations in the carnitine-acylcarnitine translocase (CACT) gene in patients with CACT deficiency and in healthy individuals. *J. Hum. Genet.* **58**, 788–793

108. Wang, G., Wang, J., Douglas, G., Browning, M., Hahn, S., Ganesh, J., Cox, S., Aleck, K., Schmitt, E. S., Zhang, W., and Wong, L.-J. C. (2011) Expanded molecular features of carnitine acyl-carnitine translocase (CACT) deficiency by comprehensive molecular analysis. *Mol. Genet. Metab.* **103**, 349–357

109. Iacobazzi, V., Invernizzi, F., Baratta, S., Pons, R., Chung, W., Garavaglia, B., Dionisi-Vici, C., Ribes, A., Parini, R., Huertas, M. D., Roldan, S., Lauria, G., Palmieri, F., and Taroni, F. (2004) Molecular and functional analysis of SLC25A20 mutations causing carnitine-acylcarnitine translocase deficiency. *Hum. Mutat.* **24**, 312–320

110. Lee, R. S. Y., Lam, C. W., Lai, C. K., Yuen, Y. P., Chan, K. Y., Shek, C. C., Chan, A. Y. W., and Chow, C. B. (2007) Carnitine-acylcarnitine translocase deficiency in three neonates presenting with rapid deterioration and cardiac arrest. *Hong Kong Med J*. **13**, 66–68

111. Huizing, M., Wendel, U., Ruitenbeek, W., Iacobazzi, V., IJlst, L., Veenhuizen, P., Savelkoul, P., van den Heuvel, L. P., Smeitink, J. A., Wanders, R. J., Trijbels, J. M., and Palmieri, F. (1998) Carnitine-acylcarnitine carrier deficiency: identification of the molecular defect in a patient. *J. Inherit. Metab. Dis.* **21**, 262–267

112. Yang, B. Z., Mallory, J. M., Roe, D. S., Brivet, M., Strobel, G. D., Jones, K. M., Ding, J. H., and Roe, C. R. (2001) Carnitine/acylcarnitine translocase deficiency (neonatal phenotype): successful prenatal and postmortem diagnosis associated with a novel mutation in a single family. *Mol. Genet. Metab.* **73**, 64–70

113. Korman, S. H., Pitt, J. J., Boneh, A., Dweikat, I., Zater, M., Meiner, V., Gutman, A., and Brivet, M. (2006) A novel SLC25A20 splicing mutation in patients of different ethnic origin with neonatally lethal carnitine-acylcarnitine translocase (CACT) deficiency. *Mol. Genet. Metab.* **89**, 332–338

114. Costa, C., Costa, J. M., Nuoffer, J. M., Slama, A., Boutron, A., Saudubray, J. M., Legrand, A., and Brivet, M. (1999) Identification of the molecular defect in a severe case of carnitine-acylcarnitine carrier deficiency. *J. Inherit. Metab. Dis.* **22**, 267–270

115. Rubio-Gozalbo, M. E., Vos, P., Forget, P. P., Van Der Meer, S. B., Wanders, R. J. A., Waterham, H. R., and Bakker, J. A. (2003) Carnitine-acylcarnitine translocase deficiency: case report and review of the literature. *Acta Paediatr.* **92**, 501–504

116. Huizing, M., Iacobazzi, V., Ijlst, L., Savelkoul, P., Ruitenbeek, W., van den Heuvel, L., Indiveri, C., Smeitink, J., Trijbels, F., Wanders, R., and Palmieri, F. (1997) Cloning of the human carnitine-

acylcarnitine carrier cDNA and identification of the molecular defect in a patient. *Am. J. Hum. Genet.* **61**, 1239–1245

117. Ij 1st, L., Ruiter, J. P., Oostheim, W., Niezen-Koning, K. E., Palmieri, F., and Wanders, R. J. (1999) Identification of a missense mutation in a patient with lethal carnitine acyl-carnitine carrier deficiency. *Adv. Exp. Med. Biol.* **466**, 347–351

118. Al Aqeel, A. I., Rashid, M. S., Ruiter, J. P., Ijlst, L., and Wanders, R. J. (2003) A novel molecular defect of the carnitine acylcarnitine translocase gene in a Saudi patient. *Clin. Genet.* **64**, 163–165

119. Galron, D., Birk, O. S., Kazanovitz, A., Moses, S. W., and Hershkovitz, E. (2004) Carnitine-acylcarnitine translocase deficiency: identification of a novel molecular defect in a Bedouin patient. *J. Inherit. Metab. Dis.* **27**, 267–273

120. Boczonadi, V., King, M. S., Smith, A. C., Olahova, M., Bansagi, B., Roos, A., Eyassu, F., Borchers, C., Ramesh, V., Lochmüller, H., Polvikoski, T., Whittaker, R. G., Pyle, A., Griffin, H., Taylor, R. W., Chinnery, P. F., Robinson, A. J., Kunji, E. R. S., and Horvath, R. (2018) Mitochondrial oxodicarboxylate carrier deficiency is associated with mitochondrial DNA depletion and spinal muscular atrophy-like disease. *Genet. Med.* **20**, 1224-1235

121. Lemattre, C., Imbert-Bouteille, M., Gatinois, V., Benit, P., Sanchez, E., Guignard, T., Tran Mau-Them, F., Haquet, E., Rivier, F., Carme, E., Roubertie, A., Boland, A., Lechner, D., Meyer, V., Thevenon, J., Duffourd, Y., Rivière, J.-B., Deleuze, J.-F., Wells, C., Molinari, F., Rustin, P., Blanchet, P., and Geneviève, D. (2019) Report on three additional patients and genotype-phenotype correlation in SLC25A22-related disorders group. *Eur. J. Hum. Genet.* **27**, 1692-1700

122. Reid, E. S., Williams, H., Anderson, G., Benatti, M., Chong, K., James, C., Ocaka, L., GOSgene, Hemingway, C., Little, D., Brown, R., Parker, A., Holden, S., Footitt, E., Rahman, S., Gissen, P., Mills, P. B., and Clayton, P. T. (2017) Mutations in SLC25A22: hyperprolinaemia, vacuolated fibroblasts and presentation with developmental delay. *J. Inherit. Metab. Dis.* **40**, 385–394

123. Giacomini, T., Pisciotta, L., Prato, G., Meola, I., Zara, F., Fiorillo, C., Baratto, S., Severino, M., De Grandis, E., and Mancardi, M. M. (2019) Severe early-onset developmental and epileptic encephalopathy (DEE) associated with novel compound heterozygous pathogenic variants in SLC25A22: Case report and literature review. *Seizure*. **70**, 56–58

124. Poduri, A., Heinzen, E., Chitsazzadeh, V., Lasorsa, F., LaCoursiere, C., Martin, E., Yusakaitis, C., Hill, R., Elhosary, P., Atabay, K., Barry, B., Partlow, J., Bashiri, F., Zeidan, R., Elmalik, S., Kabiraj, M., Kothare, S., Stödberg, T., Scheffer, I., Barkovich, A., Palmieri, F., Goldstein, D., Salih, M., and Walsh, C. (2013) SLC25A22 is a novel gene for migrating partial seizures in infancy. *Annals of Neurology*. **76**, 873–882

125. Molinari, F., Raas-Rothschild, A., Rio, M., Fiermonte, G., Encha-Razavi, F., Palmieri, L., Palmieri, F., Ben-Neriah, Z., Kadhom, N., Vekemans, M., Attie-Bitach, T., Munnich, A., Rustin, P., and Colleaux, L. (2005) Impaired mitochondrial glutamate transport in autosomal recessive neonatal myoclonic epilepsy. *Am. J. Hum. Genet*. **76**, 334–9

126. Cohen, R., Basel-Vanagaite, L., Goldberg-Stern, H., Halevy, A., Shuper, A., Feingold-Zadok, M., Behar, D. M., and Straussberg, R. (2014) Two siblings with early infantile myoclonic encephalopathy due to mutation in the gene encoding mitochondrial glutamate/H+ symporter SLC25A22. *Eur. J. Paediatr. Neurol.* **18**, 801–805

127. Molinari, F., Kaminska, A., Fiermonte, G., Boddaert, N., Raas-Rothschild, A., Plouin, P., Palmieri, L., Brunelle, F., Palmieri, F., Dulac, O., Munnich, A., and Colleaux, L. (2009) Mutations in the mitochondrial glutamate carrier SLC25A22 in neonatal epileptic encephalopathy with suppression bursts. *Clinical Genetics*. **76**, 188–194

128. Ehmke, N., Graul-Neumann, L., Smorag, L., Koenig, R., Segebrecht, L., Magoulas, P., Scaglia, F., Kilic, E., Hennig, A. F., Adolphs, N., Saha, N., Fauler, B., Kalscheuer, V. M., Hennig, F., Altmüller, J., Netzer, C., Thiele, H., Nürnberg, P., Yigit, G., Jäger, M., Hecht, J., Krüger, U., Mielke, T., Krawitz, P. M., Horn, D., Schuelke, M., Mundlos, S., Bacino, C. A., Bonnen, P. E., Wollnik, B., Fischer-Zirnsak, B., and Kornak, U. (2017) De Novo Mutations in SLC25A24 Cause a Craniosynostosis Syndrome with Hypertrichosis, Progeroid Appearance, and Mitochondrial Dysfunction. *Am. J. Hum. Genet.* **101**, 833–843

129. Writzl, K., Maver, A., Kovačič, L., Martinez-Valero, P., Contreras, L., Satrustegui, J., Castori, M., Faivre, L., Lapunzina, P., van Kuilenburg, A. B. P., Radović, S., Thauvin-Robinet, C., Peterlin, B., Del Arco, A., and Hennekam, R. C. (2017) De Novo Mutations in SLC25A24 Cause a Disorder Characterized by Early Aging, Bone Dysplasia, Characteristic Face, and Early Demise. *Am. J. Hum. Genet.* **101**, 844–855

130. Rodríguez-García, M. E., Cotrina-Vinagre, F. J., Cruz-Rojo, J., Garzón-Lorenzo, L., Carnicero-Rodríguez, P., Pozo, J. S.-D., and Martínez-Azorín, F. (2018) A rare male patient with Fontaine progeroid syndrome caused by p.R217H de novo mutation in SLC25A24. *Am. J. Med. Genet. A*. **176**, 2479–2486

131. Ryu, J., Ko, J. M., and Shin, C.-H. (2019) A 9-year-old Korean girl with Fontaine progeroid syndrome: a case report with further phenotypical delineation and description of clinical course during long-term follow-up. *BMC Med. Genet.* **20**, 188

132. Kishita, Y., Pajak, A., Bolar, N. A., Marobbio, C. M. T., Maffezzini, C., Miniero, D. V., Monné, M., Kohda, M., Stranneheim, H., Murayama, K., Naess, K., Lesko, N., Bruhn, H., Mourier, A., Wibom, R., Nennesmo, I., Jespers, A., Govaert, P., Ohtake, A., Van Laer, L., Loeys, B. L., Freyer, C., Palmieri, F., Wredenberg, A., Okazaki, Y., and Wedell, A. (2015) Intra-mitochondrial Methylation Deficiency Due to Mutations in SLC25A26. *Am. J. Hum. Genet.* **97**, 761–768

133. Schiff, M., Veauville-Merllié, A., Su, C. H., Tzagoloff, A., Rak, M., Ogier de Baulny, H., Boutron, A., Smedts-Walters, H., Romero, N. B., Rigal, O., Rustin, P., Vianey-Saban, C., and Acquaviva-Bourdain, C. (2016) SLC25A32 Mutations and Riboflavin-Responsive Exercise Intolerance. *N. Engl. J. Med.* **374**, 795–797

134. Hellebrekers, D. M. E. I., Sallevelt, S. C. E. H., Theunissen, T. E. J., Hendrickx, A. T. M., Gottschalk, R. W., Hoeijmakers, J. G. J., Habets, D. D., Bierau, J., Schoonderwoerd, K. G., and Smeets, H. J. M. (2017) Novel SLC25A32 mutation in a patient with a severe neuromuscular phenotype. *Eur. J. Hum. Genet.* **25**, 886–888

135. An, W.-B., An, W.-B., Liu, C., Wan, Y., Guo, Y., Wang, S.-C., Zhang, Y.-C., and Zhu, X.-F. (2019) [Clinical features and gene mutation spectrum in children with sideroblastic anemia]. *Zhongguo Dang Dai Er Ke Za Zhi*. **21**, 1016–1021

136. Guernsey, D. L., Jiang, H., Campagna, D. R., Evans, S. C., Ferguson, M., Kellogg, M. D., Lachance, M., Matsuoka, M., Nightingale, M., Rideout, A., Saint-Amant, L., Schmidt, P. J., Orr, A., Bottomley, S. S., Fleming, M. D., Ludman, M., Dyack, S., Fernandez, C. V., and Samuels, M. E. (2009) Mutations in mitochondrial carrier family gene SLC25A38 cause nonsyndromic autosomal recessive congenital sideroblastic anemia. *Nature genetics*. **41**, 651–3

137. Kannengiesser, C., Sanchez, M., Sweeney, M., Hetet, G., Kerr, B., Moran, E., Fuster Soler, J. L., Maloum, K., Matthes, T., Oudot, C., Lascaux, A., Pondarré, C., Sevilla Navarro, J., Vidyatilake, S., Beaumont, C., Grandchamp, B., and May, A. (2011) Missense SLC25A38 variations play an important role in autosomal recessive inherited sideroblastic anemia. *Haematologica*. **96**, 808–813

138. Mehri, M., Zarin, M., Ardalani, F., Najmabadi, H., Azarkeivan, A., and Neishabury, M. (2018) Novel mutations in mitochondrial carrier family gene SLC25A38, causing congenital sideroblastic anemia in Iranian families, identified by whole exome sequencing. *Blood Cells Mol. Dis.* **71**, 39–44

139. Liu, G., Guo, S., Kang, H., Zhang, F., Hu, Y., Wang, L., Li, M., Ru, Y., Camaschella, C., Han, B., and Nie, G. (2013) Mutation spectrum in Chinese patients affected by congenital sideroblastic anemia and a search for a genotype-phenotype relationship. *Haematologica*. **98**, e158-160

140. Le Rouzic, M.-A., Fouquet, C., Leblanc, T., Touati, M., Fouyssac, F., Vermylen, C., Jäkel, N., Guichard, J.-F., Maloum, K., Toutain, F., Lutz, P., Perel, Y., Manceau, H., Kannengiesser, C., and Vannier, J.-P. (2017) Non syndromic childhood onset congenital sideroblastic anemia: A report of 13 patients identified with an ALAS2 or SLC25A38 mutation. *Blood Cells Mol. Dis.* **66**, 11–18

141. Iuso, A., Alhaddad, B., Weigel, C., Kotzaeridou, U., Mastantuono, E., Schwarzmayr, T., Graf, E., Terrile, C., Prokisch, H., Strom, T. M., Hoffmann, G. F., Meitinger, T., and Haack, T. B. (2019) A Homozygous Splice Site Mutation in SLC25A42, Encoding the Mitochondrial Transporter of Coenzyme A, Causes Metabolic Crises and Epileptic Encephalopathy. *JIMD Rep.* **44**, 1–7

142. Shamseldin, H. E., Smith, L. L., Kentab, A., Alkhalidi, H., Summers, B., Alsedairy, H., Xiong, Y., Gupta, V. A., and Alkuraya, F. S. (2015) Mutation of the mitochondrial carrier SLC25A42 causes a novel form of mitochondrial myopathy in humans. *Hum. Genet.* 10.1007/s00439-015-1608-8

143. Almannai, M., Alasmari, A., Alqasmi, A., Faqeih, E., Al Mutairi, F., Alotaibi, M., Samman, M. M., Eyaid, W., Aljadhai, Y. I., Shamseldin, H. E., Craigen, W., and Alkuraya, F. S. (2018) Expanding the phenotype of SLC25A42-associated mitochondrial encephalomyopathy. *Clin. Genet.* **93**, 1097–1102

144. Wan, J., Steffen, J., Yourshaw, M., Mamsa, H., Andersen, E., Rudnik-Schöneborn, S., Pope, K., Howell, K. B., McLean, C. A., Kornberg, A. J., Joseph, J., Lockhart, P. J., Zerres, K., Ryan, M. M., Nelson, S. F., Koehler, C. M., and Jen, J. C. (2016) Loss of function of SLC25A46 causes lethal congenital pontocerebellar hypoplasia. *Brain*. **139**, 2877–2890

145. Braunisch, M. C., Gallwitz, H., Abicht, A., Diebold, I., Holinski-Feder, E., Van Maldergem, L., Lammens, M., Kovács-Nagy, R., Alhaddad, B., Strom, T. M., Meitinger, T., Senderek, J., Rudnik-Schöneborn, S., and Haack, T. B. (2018) Extension of the phenotype of biallelic loss-of-function mutations in SLC25A46 to the severe form of pontocerebellar hypoplasia type I. *Clin. Genet.* **93**, 255–265

146. Abrams, A. J., Hufnagel, R. B., Rebelo, A., Zanna, C., Patel, N., Gonzalez, M. A., Campeanu, I. J., Griffin, L. B., Groenewald, S., Strickland, A. V., Tao, F., Speziani, F., Abreu, L., Schüle, R., Caporali, L., La Morgia, C., Maresca, A., Liguori, R., Lodi, R., Ahmed, Z. M., Sund, K. L., Wang, X., Krueger, L. A., Peng, Y., Prada, C. E., Prows, C. A., Schorry, E. K., Antonellis, A., Zimmerman, H. H., Abdul-Rahman, O. A., Yang, Y., Downes, S. M., Prince, J., Fontanesi, F., Barrientos, A., Németh, A. H., Carelli, V., Huang, T., Zuchner, S., and Dallman, J. E. (2015) Mutations in SLC25A46, encoding a UGO1-like protein, cause an optic atrophy spectrum disorder. *Nat. Genet.* **47**, 926–932

147. Nguyen, M., Boesten, I., Hellebrekers, D. M. E. I., Mulder-den Hartog, N. M., de Coo, I. F. M., Smeets, H. J. M., and Gerards, M. (2017) Novel pathogenic SLC25A46 splice-site mutation causes an optic atrophy spectrum disorder. *Clin. Genet.* **91**, 121–125

148. Charlesworth, G., Balint, B., Mencacci, N. E., Carr, L., Wood, N. W., and Bhatia, K. P. (2016) SLC25A46 mutations underlie progressive myoclonic ataxia with optic atrophy and neuropathy. *Mov. Disord.* **31**, 1249–1251

149. Janer, A., Prudent, J., Paupe, V., Fahiminiya, S., Majewski, J., Sgarioto, N., Des Rosiers, C., Forest, A., Lin, Z.-Y., Gingras, A.-C., Mitchell, G., McBride, H. M., and Shoubridge, E. A. (2016) SLC25A46 is required for mitochondrial lipid homeostasis and cristae maintenance and is responsible for Leigh syndrome. *EMBO Mol Med*. **8**, 1019–1038

150. Hammer, M. B., Ding, J., Mochel, F., Eleuch-Fayache, G., Charles, P., Coutelier, M., Gibbs, J. R., Arepalli, S. K., Chong, S. B., Hernandez, D. G., Majounie, E., Clipman, S., Bouhlal, Y., Nehdi, H., Brice, A., Hentati, F., Stevanin, G., Amouri, R., Durr, A., and Singleton, A. B. (2017) SLC25A46 Mutations Associated with Autosomal Recessive Cerebellar Ataxia in North African Families. *Neurodegener Dis*. **17**, 208–212

151. Abrams, A. J., Fontanesi, F., Tan, N. B. L., Buglo, E., Campeanu, I. J., Rebelo, A. P., Kornberg, A. J., Phelan, D. G., Stark, Z., and Zuchner, S. (2018) Insights into the genotype-phenotype correlation and molecular function of SLC25A46. *Hum. Mutat.* **39**, 1995–2007

152. Sulaiman, R. A., Patel, N., Alsharif, H., Arold, S. T., and Alkuraya, F. S. (2017) A novel mutation in SLC25A46 causes optic atrophy and progressive limb spasticity, with no cerebellar atrophy or axonal neuropathy. *Clin. Genet.* **92**, 230–231
